# A Comprehensive Evaluation of Steroid Metabolism in Women with Intrahepatic Cholestasis of Pregnancy

**DOI:** 10.1371/journal.pone.0159203

**Published:** 2016-08-05

**Authors:** Antonín Pařízek, Martin Hill, Michaela Dušková, Libor Vítek, Marta Velíková, Radmila Kancheva, Patrik Šimják, Michal Koucký, Zuzana Kokrdová, Karolína Adamcová, Andrej Černý, Zdeněk Hájek, Luboslav Stárka

**Affiliations:** 1 Department of Obstetrics and Gynecology, General University Hospital and 1st Faculty of Medicine, Charles University in Prague, Prague, Czech Republic; 2 Institute of Endocrinology, Prague, Czech Republic; 3 Institute of Medical Biochemistry and Laboratory Diagnostics and 4th Department of Internal Medicine, General University Hospital and 1st Faculty of Medicine of Charles University in Prague, Prague, Czech Republic; RWTH Aachen, GERMANY

## Abstract

Intrahepatic cholestasis of pregnancy (ICP) is a common liver disorder, mostly occurring in the third trimester. ICP is defined as an elevation of serum bile acids, typically accompanied by pruritus and elevated activities of liver aminotransferases. ICP is caused by impaired biliary lipid secretion, in which endogenous steroids may play a key role. Although ICP is benign for the pregnant woman, it may be harmful for the fetus. We evaluated the differences between maternal circulating steroids measured by RIA (17-hydroxypregnenolone and its sulfate, 17-hydroxyprogesterone, and cortisol) and GC-MS (additional steroids), hepatic aminotransferases and bilirubin in women with ICP (n = 15, total bile acids (TBA) >8 μM) and corresponding controls (n = 17). An age-adjusted linear model, receiver-operating characteristics (ROC), and multivariate regression (a method of orthogonal projections to latent structure, OPLS) were used for data evaluation. While aminotransferases, conjugates of pregnanediols, 17-hydroxypregnenolone and 5β-androstane-3α,17β-diol were higher in ICP patients, 20α-dihydropregnenolone, 16α-hydroxy-steroids, sulfated 17-oxo-C19-steroids, and 5β-reduced steroids were lower. The OPLS model including steroids measured by GC-MS and RIA showed 93.3% sensitivity and 100% specificity, while the model including steroids measured by GC-MS in a single sample aliquot showed 93.3% sensitivity and 94.1% specificity. A composite index including ratios of sulfated 3α/β-hydroxy-5α/β-androstane-17-ones to conjugated 5α/β-pregnane-3α/β, 20α-diols discriminated with 93.3% specificity and 81.3% sensitivity (ROC analysis). These new data demonstrating altered steroidogenesis in ICP patients offer more detailed pathophysiological insights into the role of steroids in the development of ICP.

## Introduction

Intrahepatic cholestasis of pregnancy (ICP) is the most common liver disorder in pregnancy, with incidence depending on geographic location. In most countries of Europe and Northern America the incidence ranges between 0.4 to 1% [1]. ICP (commonly inducing placental oxidative stress and apoptosis [2]) is most probably associated with an altered biliary secretion of bile acids (BA) and other biliary lipids. Although ICP is an unpleasant condition for the pregnant woman, it does not represent a serious threat. In contrast, ICP raises the risk of serious and difficult-to-predict complications in the fetus including preterm birth, meconium flux into the amniotic fluid, respiratory distress syndrome, and sudden intrauterine fetal death [3].

The risk of ICP increases with increasing levels of pregnancy steroids, which reflect the advancing gestational age (GA). Furthermore, the risk of ICP is higher in multiple pregnancies, with multiple sources of fetal steroid precursors such as sulfated C21 and C19 Δ^5^ steroids. These substances penetrate into the placenta, where they are converted into unconjugated equivalents and consequently to progestogens and estrogens, respectively. Some of the progestogens may be converted to their 5α-reduced metabolites, which may be subsequently sulfated and/or glucuronized in the maternal liver. In addition, 5α-reduced metabolites of fetal origin as well as 5β-reduced-pregnanes primarily originating in the fetal liver may also penetrate from the fetus to the maternal circulation across the placenta, forming steroid conjugates in the maternal liver. As we have previously reported, the conjugation of 5α/β-reduced-20-oxo-pregnanes in the maternal compartment increases with approaching term [4,5].

In addition, ICP symptoms disappear shortly after birth as the placental conversion of fetal steroids disappears and the source of fetal steroids in the fetal adrenal zone gradually fades [6–9]. As suggested by several authors, ICP is probably associated with an overproduction of some sulfated progesterone metabolites such as pregnanolone isomers and pregnanediols [7,9–12]. Some studies also indicate that a key role in the pathophysiology of ICP may be mediated by enzymes converting the 3β-hydroxy and 3-oxo-progesterone catabolites to their 3α-hydroxy equivalents, while others suggest a selective defect in steroid sulfate conjugation affecting the biliary secretion of steroid metabolites [7]. The importance of progesterone catabolism in the pathophysiology of ICP is further supported by reduced progesterone levels, while elevated concentrations of its metabolites are found in women with asymptomatic hypercholanemia (moderately elevated TBA (>11 μM) but normal aminotransferases) [13].

BAs are potent ligands of the hepatic farnesoid X receptor (FXR), which protects the liver from potentially harmful effects due to their accumulation leading to inhibition of their *de novo* synthesis [14]. As the progesterone catabolite 3β,5α-THP sulfate inhibits the FXR [15], the overproduction of conjugated progesterone catabolites may also induce the development of ICP *via* this pathway. FXR functioning may be regulated by estrogen receptor α (ER-α), the activation of which inhibits the FXR and consequently stimulates the expression of pro-cholestatic genes [16]. Some experiments on animal models have indicated the detrimental effects of estradiol and of reduced progesterone metabolites. These steroids inhibit the bile salt export pump (BSEP), which is responsible for BA secretion. In contrast to its reduced metabolites, progesterone itself is inactive [17,18]. Nevertheless, concerning the associations of estrogens and dehydroepiandrosterone sulfate (DHEAS) with ICP, the data in the literature are equivocal [19–21]. Regarding the adverse effects of BA on steroidogenesis, Martineau *et al*. [22] found that elevated levels of circulating BA attenuate the expression of protective type 2 11β-hydroxysteroid dehydrogenase (HSD11B2), which restrains the influx of high amounts of cortisol from the maternal compartment into the fetus.

All these data indicate a key role for steroids in the development of ICP. However, a number of questions and controversies remain. In this study, we mapped the alterations in maternal circulating steroids to evaluate the associations of individual steps of steroid metabolism with the risk of ICP, and to assess the potential importance of maternal circulating steroids for the diagnosis of ICP. For this purpose, we attempted to build a predictive model discriminating patients with ICP from controls based on steroids in the maternal serum measured by CG-MS in a single sample aliquot.

## Materials and Methods

### Subjects

Fifteen pregnant women with ICP were included in the study. The mean gestational age at the onset of ICP was 35.4 ± 2.5 weeks (range 29–39). All women with ICP had increased TBA levels (≥ 8 μmol/l), 82% of them exhibited elevated aminotransferases and 54% of them suffered from pruritus. Other causes of cholestasis were excluded by detailed clinical and laboratory examinations. Eight cases of severe ICP (TBA > 40 μmol/l) were recorded. All pregnancies were singleton. The control group consisted of 17 women with uncomplicated singleton pregnancies, who underwent laboratory examination in the 36^th^ week. No case of maternal pruritus was noted and serum aminotransferase activities and TBA were normal in all controls. The mean gestational age at delivery among patients with ICP and controls were 37.3 ± 1.0 and 39.3 ± 1.2, respectively. Pregnancy ended prematurely in 3 cases of ICP (20%), whereas in the control group no case of delivery before the 37^th^ week of pregnancy occurred. From the total of ICP-complicated pregnancies, 54% were terminated by cesarean section, in contrast to only 24% in the control group. The mean APGAR score at the first minute was 9.2 ± 0.7 and 9.2 ± 1.1, and at fifth minute was 10.0 ± 0.0 and 9.7 ± 0.8 for the ICP and control groups, respectively.

The ethics committee of the General University Hospital and 1^st^ Faculty of Medicine of Charles University in Prague approved this study. After obtaining approval from the ethical committee of our institution, written informed consent was obtained from all patients. Once participants met inclusion criteria, they were instructed in detail about the purpose and process of the study. They were given sufficient time to discuss the study with their doctor and to decide whether to participate or not.

### Sample collection

Blood samples were collected before the beginning of the treatment by UDCA. Serum was obtained after centrifugation for 5 min at 2000×g at 0°C., and stored at −20°C until analyzed.

### Chemicals

Steroids were purchased from Steraloids (Newport, RI, USA), Sylon B from Supelco (Bellefonte, PA, USA), methoxylamine hydrochloride from Sigma (St. Louis, MO, USA) and solvents from Merck (Darmstadt, Germany).

### Instruments and chromatography conditions

A GCMS-QP2010 Plus system from Shimadzu (Kyoto, Japan) consisting of a gas chromatograph equipped with automatic flow control, an AOC-20s autosampler and a single quadrupole detector with an adjustable electron voltage of 10–195 V was utilized. A capillary column with a medium polarity RESTEK Rxi column (diameter 0.25mm, length 15 m, film thickness 0.1 μm) was used for analyses. Electron-impact ionization with electron voltage fixed at 70V and emission current set to 160 μA was used for the measurements. The temperatures of the injection port, ion source and interface were maintained at 220, 300, and 310°C, respectively. Analyses were carried out in the splitless mode with a constant linear velocity of the carrier gas (He), which was maintained at 60 cm/s. The septum purge flow was set to 3 mL/min. The samples were injected using the high pressure mode, which was applied at 200 kPa and maintained for 1 min. The detector voltage was set to 1.4 kV.

### Analytical methods

#### Liver function tests

Standard serum biochemical markers such as alanine aminotransferase (ALT), aspartate aminotransferase (AST), γ-glutamyltransferase (GGT), alanine aminotransferase (ALP) and bilirubin were determined on an automatic analyzer (Modular analyzer, Roche Diagnostics GmbH, Mannheim, Germany), using routine laboratory assays. TBAs were quantified using an EIA kit from Trinity Biotech USA Inc. (Jamestown, NY, USA).

#### Instruments and chromatography conditions

In total, the levels of 63 analytes were quantified in the circulation of women with ICP and in corresponding controls. These analytes included a group of liver function tests, 38 unconjugated steroids and 25 steroid conjugates. The steroid metabolome in the maternal circulation included the levels of C21 Δ^5^ steroids, C19 Δ^5^ steroids, C21 Δ^4^ steroids, C19 Δ^4^ steroids, estrogens, C21 5α/β-reduced steroids and C19 5α/β-reduced steroids. Most of the steroids were measured by GC-MS using our previously published method (for details see [23–25]); however, 17-hydroxypregnenolone and 17-hydroxypregnenolone sulfate were measured by our previously published RIA methods [26,27], while 17-hydroxyprogesterone, cortisol, testosterone, and sex hormone binding globulin (SHBG) were quantified by RIA kits from Immunotech (Marseille, France) (for the steroids including their abbreviations see Table 1).

**Table 1:** Steroids quantified in the circulation of women with ICP and controls.

**C21 Δ**^**5**^ **Steroids**	Estradiol sulfate (GC-MS)
Pregnenolone (GC-MS)	16α-Hydroxyestrone (GC-MS)
Pregnenolone sulfate (GC-MS)	16α-Hydroxyestrone sulfate (GC-MS)
20α-Dihydropregnenolone (GC-MS)	Estriol (GC-MS)
20α-Dihydropregnenolone sulfate (GC-MS)	Estriol sulfate (GC-MS)
17-Hydroxypregnenolone (RIA)	
17-Hydroxypregnenolone sulfate (RIA)	**C21 5α/β-reduced steroids**
16α-Hydroxypregnenolone (GC-MS)	5α-Dihydroprogesterone (GC-MS)
	Allopregnanolone (3α,5α-THP) (GC-MS)
**C19 Δ**^**5**^ **Steroids**	Allopregnanolone sulfate (GC-MS)
DHEA (GC-MS)	Isopregnanolone (3β,5α-THP) (GC-MS)
DHEA sulfate (GC-MS)	Isopregnanolone sulfate (GC-MS)
7α-Hydroxy-DHEA (GC-MS)	5β-Dihydroprogesterone (GC-MS)
7β-Hydroxy-DHEA (GC-MS)	Pregnanolone (3α,5β-THP) (GC-MS)
16α-Hydroxy-DHEA (GC-MS)	Conjugated pregnanolone (GC-MS)
16α-Hydroxy-DHEA sulfate (GC-MS)	Epipregnanolone (3β,5β-THP) (GC-MS)
Androstenediol (GC-MS)	Conjugated epipregnanolone (GC-MS)
Androstenediol sulfate (GC-MS)	5α,20α-Tetrahydroprogesterone (GC-MS)
5-Androstene-3β,7α,17β-triol (GC-MS)	5α-Pregnane-3α,20α-diol (3α,5α,20α-PD) (GC-MS)
5-Androstene-3β,7β,17β-triol (GC-MS)	Conjugated 5α-pregnane-3α,20α-diol (GC-MS)
	5α-Pregnane-3β,20α-diol (3β,5α,20α-PD) (GC-MS)
**C21 Δ**^**4**^ **Steroids**	Conjugated 5α-pregnane-3β,20α-diol (GC-MS)
Progesterone (GC-MS)	5β,20α-Tetrahydroprogesterone (GC-MS)
20α-Dihydroprogesterone (GC-MS)	5β-Pregnane-3α,20α-diol (3α,5β,20α-PD) (GC-MS)
17-Hydroxyprogesterone (RIA)	Conjugated 5β-pregnane-3α,20α-diol (GC-MS)
16α-Hydroxyprogesterone (GC-MS)	Conjugated 5β-pregnane-3β,20α-diol (conjugated 3β,5β,20α-PD) (GC-MS)
Cortisol (RIA)	
	**C19 5α/β-reduced steroids**
**C19 Δ**^**4**^ **Steroids**	Androsterone (3α,5α-THA) (GC-MS)
Androstenedione (GC-MS)	Androsterone sulfate (GC-MS)
16α-Hydroxyandrostenedione (GC-MS)	Epiandrosterone (3β,5α-THA) (GC-MS)
Testosterone (RIA)	Epiandrosterone sulfate (GC-MS)
16α-Hydroxytestosterone (GC-MS)	Etiocholanolone (3α,5β-THA) (GC-MS)
	Etiocholanolone sulfate (GC-MS)
**Estrogens**	Epietiocholanolone (3β,5β-THA) sulfate (GC-MS)
Estrone (GC-MS)	Conjugated 5α-androstane-3α,17β-diol (3α,5α,17β-AD) (GC-MS)
Estrone sulfate (GC-MS)	Conjugated 5α-androstane-3β,17β-diol (3β,5α,17β-AD) (GC-MS)
Estradiol (GC-MS)	Conjugated 5β-androstane-3α,17β-diol (3α,5β,17β-AD) (GC-MS)

#### Sample preparation and GC-MS analysis

The sample preparation for GC-MS analysis proceeded as follows: unconjugated steroids were extracted from 1 mL of serum fluid with diethyl-ether (3 mL). The diethyl-ether extract was then dried in a block heater at 37°C, and the lipids in the dry residue of this extract were separated by partitioning between a mixture of methanol–water 4:1 (1 mL) and pentane (1 mL). The pentane phase was discarded and the polar phase was dried in a vacuum centrifuge at 60°C (2 h). The dry residue from the polar phase was derivatized first with methoxylamine hydrochloride solution in pyridine (2%) on oxo-groups (60°C, 1 h). After the first derivatization this mixture was dried in a flow of nitrogen and the dry residue was treated with the reagent Sylon B (99% bis(trimethylsilyl)-trifluoroacetamide and 1% trimethylchlorosilane), forming trimethylsilyl derivatives on hydroxy-groups (TMS-MOX derivatives) (90°C, 1 h). Finally, after the second derivatization step the mixture was dried in a flow of nitrogen, the dry residue was dissolved in 20 μL of isooctane, and 1 μL of this solution was used for GC–MS analysis. Steroid conjugates remaining in the polar residues after diethyl ether extraction were analyzed as follows: the polar residues were dried in a vacuum centrifuge at 37°C (5 h) and the dry residues were hydrolyzed as described elsewhere [28]. The hydrolyzed samples were again dried in the vacuum centrifuge at 37°C (5 h). The dried residues were reconstituted with 1 mL of chromatographic water and then further processed in the same way as the free steroids. In contrast to the sample preparation of free steroids, the dry residue after the second derivatization step was dissolved in 200 μL instead of 20 μL of isooctane. Prior to further processing, the original samples and the polar phases after diethyl-ether extraction, which were used for the quantification of the steroid conjugates, were spiked with 17α-estradiol (as an internal standard) to attain concentrations of 1 and 10 ng/mL, respectively. The internal standard was recorded at the effective masses m/z = 231, 285 and 416.

#### Terminology of steroid polar conjugates

Concerning the terminology of the steroid polar conjugates used here, the term steroid sulfate was used in the case of the dominance of 3α/β-monosulfate over other forms of steroid conjugates, while the term conjugated steroid was used in the case of comparable amounts of conjugate forms (sulfates, disulfates, and glucuronides). This terminology was based on the relevant literature, with appropriate citations for each steroid as follows: pregnenolone sulfate [29,30], 20α-dihydropregnenolone sulfate, DHEAS [30–32], 16α-hydroxy-DHEA sulfate [30,33], androstenediol sulfate [30,31], 3α,5α-THP sulfate [11], 3β,5α-THP sulfate [15], conjugated 3α,5β-THP (sulfate + glucuronide [11]), 5α,3β,20α-PD sulfate (3β,20α-disulfate + 3β-sulfate) [11], conjugated 3α,5β,20α-PD (3β,20α-disulfate + 3β-sulfate + glucuronide) [11], 3α,5α-THA sulfate [30,31], 3β,5α-THA sulfate [30,31], 3α,5β-THA sulfate [33], 3β,5β-THA sulfate, conjugated 5α-androstane-3α,17β-diol (glucuronide + sulfate [31]), and conjugated 5α-androstane-3β,17β-diol (sulfate + glucuronide [31]).

### Calculations and data analysis

#### Composite index of ratios of sulfated 3α/β-hydroxy-5α/β-androstane-17-ones to conjugated 5α/β-pregnane-3α/β, 20α-diols for discrimination of ICP patients from controls

Taking into account the generally suppressed levels of sulfated 3α/β-hydroxy-5α/β-androstane-17-ones but elevated levels of conjugated 5α/β-pregnane-3α/β, 20α-diols (as will be discussed later in the text), we suggested a composite index *I*_*A/P*_ defined by the expression as follows:

$$I_{A/P}=\sqrt[{10}]{\frac{\left[3\alpha ,5\alpha -THA,S\right]}{\left[3\alpha ,5\alpha ,20\alpha -PD,C\right]}\cdot \frac{\left[3\beta ,5\alpha -THA,S\right]}{\left[3\beta ,5\alpha ,20\alpha -PD,C\right]}\cdot \frac{\left[3\alpha ,5\beta -THA,S\right]}{\left[3\alpha ,5\beta ,20\alpha -PD,C\right]}\cdot \frac{\left[3\beta ,5\beta -THA,S\right]}{\left[3\beta ,5\beta ,20\alpha -PD,C\right]}}$$ (1)

where [3*α*,5*α*−*THA*,*S*], [3*β*,5*α*−*THA*,*S*], [3*β*,5*α*−*THA*,*S*], [3*α*,5*β*−*THA*,*S*], and [3*β*,5*β*−*THA*,*S*] are the concentrations of sulfated androsterone, epiandrosterone, etiocholanolone, and epietiocholanolone, respectively and [3*α*,5*α*,20*α*−*PD*,*C*], [3*β*,5*α*,20*α*−*PD*,*C*], [3*α*,5*β*,20*α*−*PD*,*C*], and [3*β*,5*β*,20*α*−*PD*,*C*] are the concentrations of conjugated 5α-pregnan-3α,20α-diol, 5α-pregnan-3β,20α-diol, 5β-pregnan-3α,20α-diol, and 5β-pregnan-3β,20α-diol, respectively (monosulfates + disulfates + glucuronides). The 10^th^ root was used to attain a Gaussian data distribution and constant variance for *I*_*A/P*_.

#### Statistical analysis

To interpret the relationships between steroids and the presence of ICP in women, we used four statistical approaches.

**Gestational age-adjusted linear model:** The first method compared steroid levels in the ICP group and in corresponding controls. Respecting the dependence of most maternal circulating steroids on GA [5,23], we used linear models adjusted to constant GA (polynomial of the 2^nd^ degree for the dependence on GA) to separate the variability in the dependent variable shared with GA from that explained by the subjects’ ICP status (ICP positive vs. controls). Respecting the skewed distribution and non-constant variance in most dependent variables, these were transformed by power transformations to achieve data symmetry and homoscedasticity prior to further data processing [34]. The homogeneity and distribution of the transformed data were checked by residual analysis as described elsewhere [35,36]. The statistical software Statgraphics Centurion, version XV from Statpoint Inc. (Herndon, Virginia, USA) was used for the linear model.

**Receiver-operating characteristic:** The second method used for data evaluation was the analysis using a receiver-operating characteristic (ROC), as explained elsewhere [37,38]. The optimum cut-off value was found using the maximum of Youden’s index for rating diagnostic tests [39]. The statistical software NCSS 2007 from Number Cruncher Statistical Systems (Kaysville, Utah, USA) was used for the ROC analysis.

**Multivariate regression with a reduction of dimensionality, orthogonal projections to latent structure (OPLS):** The third and in-the-end most efficient method was a simultaneous evaluation of relationships between steroids and the presence of ICP by multivariate regression, with a reduction of dimensionality known as orthogonal projections to latent structure (OPLS) [38,40–42]. OPLS is capable of coping with the problem of severe multicollinearity (high intercorrelations) in the matrix of predictors, while ordinary multiple regression fails to evaluate such data. The multicollinearity in OPLS is favorable as it enhances the predictivity of the model. In our OPLS models, the logarithm of the ratio of the probability that the subject is a patient (with ICP) to the probability that the subject is a control (logarithm of the likelihood ratio, LLR) was chosen as a single dependent variable, while the age of the subject and steroid levels were the predictors.

The variability in predictors was separated into two independent components. The first one contained the variability predictors, which was shared with the probability of pathology (the predictive component) while the orthogonal components explained the variability shared within the highly intercorrelated predictors.

The OPLS identified the best predictors as well as the best combination of predictors to estimate the probability of the presence of pathology. After standardization of the variables, the OPLS model can be expressed as follows:

$$X=T_{p}P_{p}^{T}+T_{0}P_{0}^{T}+E$$ (2)

$$Y=T_{p}P_{p}^{T}+F$$ (3)

where **X** is the matrix with predictors and subjects, **Y** is the vector of dependent variable and subjects; **T**_p_ is the vector of component scores from the single predictive component and subjects extracted from **Y**; **T**_o_ is the vector of component scores from the single orthogonal component and subjects extracted from **X**; **P**_p_ is the vector of component loadings for the predictive component extracted from **Y**; **P**_o_ is the vector of component loadings for the orthogonal component extracted from **X** and independent variables; and **E** and **F** are the error terms.

The relevant predictors were chosen using variable importance (VIP) statistics. The statistical software SIMCA-P v.12.0 from Umetrics AB (Umeå, Sweden), which was used for OPLS analysis, enabled finding the number of relevant components, the detection of multivariate non-homogeneities, and testing the multivariate normal distribution and homoscedasticity (constant variance).

**Fig 1:**
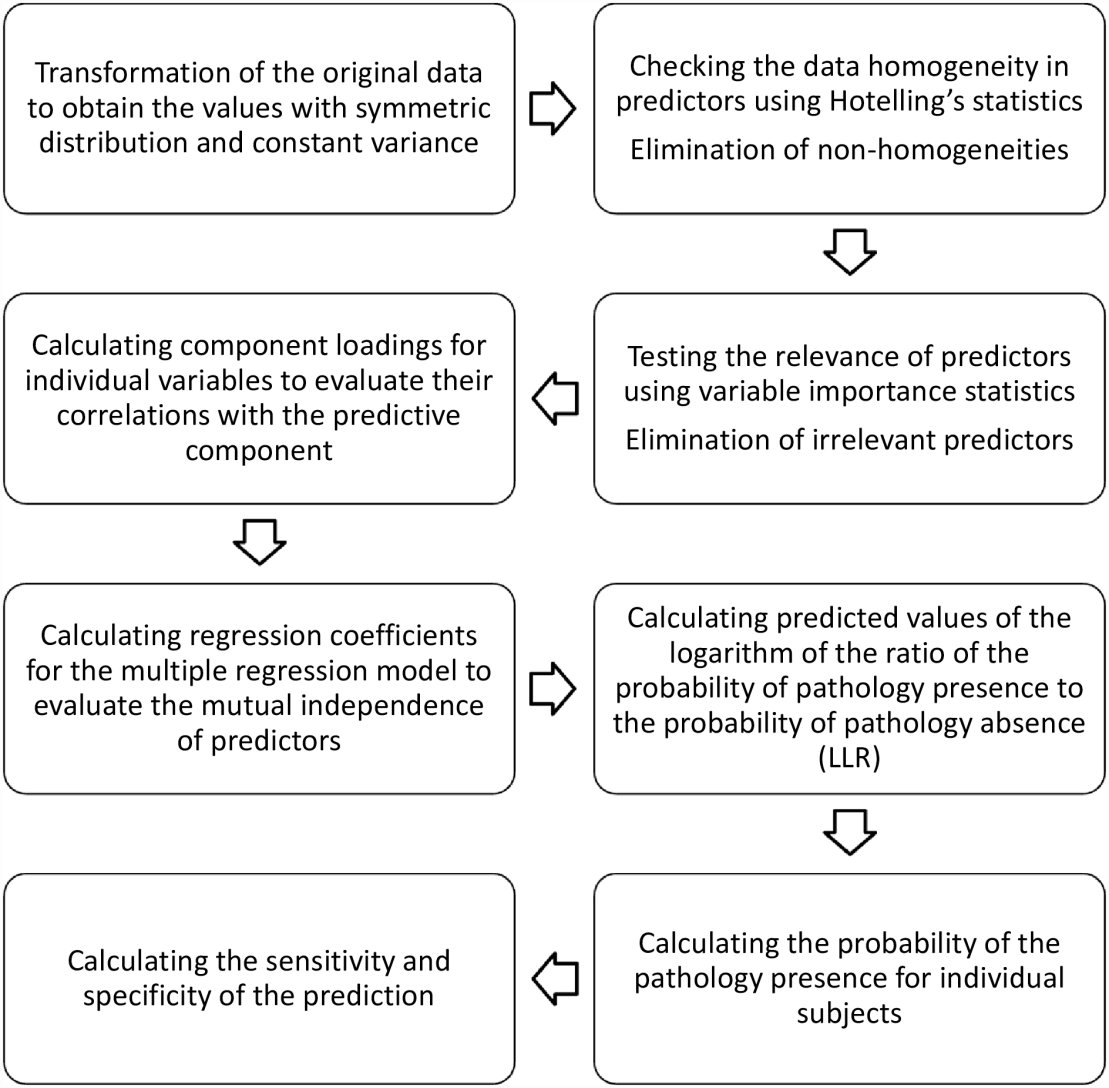
Algorithm for obtaining predictions of intrahepatic cholestasis of pregnancy (ICP).

The algorithm for obtaining the predictions is shown in Fig 1.

**Multiple regression:** The fourth method was a multiple regression without a reduction of dimensionality. This approach identified relevant predictors for which correlation with the dependent variable was independent of the remaining predictors.

## Results

Table 2 shows the relevant results of hepatic tests and steroids with significant differences (p<0.05) and differences close to statistical significance (p<0.1) between ICP patients and controls. Since having an elevated TBA level was an inclusion criterion for the ICP group, these levels were pronouncedly higher in ICP patients (F = 169, p<0.001), as expected. Similarly, the remaining liver function tests such as ALT (F = 82.5, p<0.001), AST (F = 26.7, p<0.001), GGT (F = 11.2, p = 0.003), ALP (F = 9.6, p = 0.006), and bilirubin (F = 9.0, p = 0.006) were also substantially elevated in the ICP group.

**Table 2:** Levels of relevant^a^ liver function tests and circulating steroids in patients with ICP and controls.

		Mean (lower 95%, upper 95% CI)		ICP^b^	
Variable	Unit	Controls	ICP	*F*	*p*
**Alanine aminotransferase (ALT)**	**μkat/L**	**0.274 (0.236; 0.318)**	**1.56 (1.21; 2.1)**	**82.5**	**<0.001**
**Aspartate aminotransferase (AST)**	**μkat/L**	**0.402 (0.349; 0.464)**	**1.06 (0.843; 1.39)**	**26.7**	**<0.001**
**γ-Glutamyltransferase (GGT)**	**μkat/L**	**0.182 (0.128; 0.248)**	**0.526 (0.394; 0.683)**	**11.2**	**0.003**
**Alkaline phosphatase (ALP)**	**μkat/L**	**2.26 (2; 2.54)**	**3.42 (2.99; 3.93)**	**9.6**	**0.006**
**Total bile acids (TBA)**	**μM**	**4.63 (4.1; 5.23)**	**31.6 (26.2; 39)**	**169**	**<0.001**
20α-Dihydropregnenolone	nM	1.72 (1.48; 2.02)	1.31 (1.14; 1.52)	3	0.093
17-Hydroxypregnenolone	nM	0.483 (0.309; 0.745)	1.13 (0.714; 1.76)	3.3	0.082
**17-Hydroxypregnenolone sulfate**	**nM**	**10.1 (7.89; 12.5)**	**19.2 (16.2; 22.3)**	**10.3**	**0.003**
16α-Hydroxy-DHEA	nM	0.516 (0.317; 0.848)	0.199 (0.116; 0.336)	3.2	0.085
16α-Hydroxyandrostenedione	nM	0.46 (0.328; 0.634)	0.216 (0.14; 0.32)	3.9	0.06
**16α-Hydroxyestrone**	**nM**	**0.307 (0.214; 0.427)**	**0.12 (0.0697; 0.19)**	**4.8**	**0.038**
5β-Dihydroprogesterone	nM	0.914 (0.767; 1.09)	1.28 (1.05; 1.55)	3	0.096
Pregnanolone (3α,5β-THP)	nM	11.6 (9.9; 13.3)	8.11 (6.61; 9.74)	4	0.056
**Conjugated epipregnanolone (3β,5β-THP)**	**nM**	**50.4 (38.8; 67.1)**	**25.8 (20.3; 33.1)**	**6.3**	**0.019**
**Conjugated 5α-pregnane-3α,20α-diol**	**μM**	**6.65 (4.85; 9.14)**	**16.6 (11.8; 23.6)**	**7**	**0.013**
**Androsterone (3α,5α-THA) sulfate**	**nM**	**313 (237; 415)**	**100 (73.3; 136)**	**14**	**<0.001**
**Epiandrosterone (3β,5α-THA) sulfate**	**nM**	**75.4 (58.6; 96.2)**	**35.9 (26.4; 47.9)**	**6.9**	**0.014**
**Epietiocholanolone (3β,5β-THA) sulfate**	**nM**	**5.9 (4.4; 7.91)**	**2.08 (1.48; 2.88)**	**10.5**	**0.003**
**Conjugated 5α-androstane-3α,17β-diol**	**nM**	**12.5 (10.1; 15.7)**	**22.4 (17; 31)**	**4.9**	**0.036**
Conjugated 5β-androstane-3α,17β-diol	nM	1.29 (0.899; 1.86)	2.76 (1.84; 4.29)	3.6	0.07
**3α,5α,20α-PD/3α,5α-THA, conjugates**		**23 (16.4; 32.1)**	**123 (81.9; 192)**	**19.4**	**<0.001**
**3β,5α,20α-PD/3β,5α-THA, conjugates**		**204 (162; 259)**	**642 (475; 895)**	**16.9**	**<0.001**
**3α,5β,20α-PD/3α,5β-THA, conjugates**		**142 (113; 179)**	**325 (248; 436)**	**10.1**	**0.004**
**3β,5β,20α-PD/3β,5β-THA, conjugates**		**170 (132; 222)**	**542 (386; 794)**	**13.6**	**0.001**
^c^***I***_***A/P***_		**0.159 (0.147; 0.172)**	**0.097 (0.0841; 0.11)**	**21.9**	**<0.001**

^a^Only those variables with differences between patients and controls at a significance levels p<0.1 are shown;

^b^a gestational age-adjusted linear model (polynomial of the 2^nd^ degree for the gestational age dependence) was used for the evaluation of differences between controls and patients;

^c^a composite index of ratios of sulfated 3α/β-hydroxy-5α/β-androstane-17-ones to conjugated 5α/β-pregnane-3α/β, 20α-diols (for details see the section Calculations and data analysis)

Several steroids such as 17-hydroxypregnenolone sulfate (F = 10.3, p = 0.003), conjugated 3α,5α,20α-PD (F = 7.0, p = 0.013), and conjugated 3α,5α,17β-AD (F = 4.9, p = 0.036) were significantly higher in the ICP patients, while the opposite was found for 17-oxo C19 5α-reduced metabolites such as 3α,5α-THA (F = 14, p<0.001), 3β,5α-THA (F = 6.9, p = 0.014), and 5β-reduced steroids such as 3β,5β-THP (F = 6.3, p = 0.019) and 3β,5β-THA (F = 10.5, p = 0.003).

The discrimination between ICP patients and controls by receiver operating characteristics (ROC) with a significant area under the curve (AUC) (p<0.05) or AUC close to statistical significance (p<0.1) is shown in Table 3. As expected, in addition to TBA, which was used as the gold standard for discrimination between ICP patients and controls (for patients TBA > 8 μM), most of the remaining liver function tests were also higher in ICP patients. Of these analytes, the efficiency of discrimination decreased in the sequence: ALT, bilirubin, AST, and GGT, while the AUC for ALP was not significant.

**Table 3:** Discrimination between women with ICP and age- and gender-corresponding controls on the basis of relevant^a^ liver function tests and circulating steroids measured by GC-MS or RIA as evaluated by receiver operating characteristics (ROC).

Variable	Unit	Cut-off value (patient)	Sensitivity	Specificity	AUC	p-value (2-sided)^b^
**Alanine aminotransferase (ALT)**	**μkat/L**	**>0.81**	**92.3**	**100**	**0.923**	**<0.001**
**Aspartate aminotransferase (AST)**	**μkat/L**	**>0.68**	**76.9**	**100**	**0.846**	**<0.001**
**γ-Glutamyltransferase (GGT)**	**μkat/L**	**>0.39**	**69.2**	**93.8**	**0.829**	**<0.001**
**20α-Dihydropregnenolone**	**nM**	**<1.51**	**86.7**	**58.8**	**0.722**	**0.017**
**17-Hydroxypregnenolone sulfate**	**nM**	**>18.1**	**66.7**	**94.1**	**0.776**	**0.002**
**16α-Hydroxy-DHEA**	**nM**	**<0.56**	**86.7**	**64.7**	**0.737**	**0.011**
**16α-Hydroxyandrostenedione**	**nM**	**<0.21**	**53.3**	**88.2**	**0.694**	**0.044**
16α-Hydroxyestrone	nM	<0.26	80	70.6	0.678	0.081
Allopregnanolone (3α5α-THP) sulfate	nM	>1500	60	75	0.671	0.089
**Pregnanolone (3α5β-THP)**	**nM**	**<11.2**	**86.7**	**70.6**	**0.79**	**<0.001**
**Epipregnanolone (3α5β-THP)**	**nM**	**<0.45**	**60**	**94.1**	**0.769**	**0.003**
**Conjugated 5α-pregnane-3α,20α-diol**	**nM**	**>7050**	**93.3**	**56.3**	**0.831**	**<0.001**
Conjugated 5α-pregnane-3β,20α-diol	μM	>26.7	60	81.3	0.681	0.075
5β,20α-Tetrahydroprogesterone	nM	<0.74	60	76.5	0.675	0.088
Conjugated 5β-pregnane-3α,20α-diol	nM	>2530	53.3	81.3	0.675	0.082
Androsterone (3α5α-THA) sulfate	nM	<129	66.7	75	0.692	0.056
**Epiandrosterone sulfate (3β5α-THA)**	**nM**	**<42**	**60**	**87.5**	**0.763**	**0.003**
**Etiocholanolone (3α5β-THA)**	**nM**	**<0.09**	**66.7**	**82.4**	**0.722**	**0.024**
**Conjugated etiocholanolone**	**nM**	**<3.95**	**53.3**	**100**	**0.717**	**0.035**
**Epietiocholanolone (3β5β-THA) sulfate**	**nM**	**<3.13**	**80**	**81.3**	**0.817**	**<0.001**
**Conjugated 5α-androstane-3α,17β-diol**	**nM**	**>14.7**	**80**	**62.5**	**0.758**	**0.003**
**3α,5α,20α-PD/3α,5α-THA, conjugates**		**>77.7**	**73.3**	**100**	**0.900**	**<0.001**
**3β,5α,20α-PD/3β,5α-THA, conjugates**		**>244**	**100**	**62.5**	**0.888**	**<0.001**
**3α,5β,20α-PD/3α,5β-THA, conjugates**		**>192**	**86.7**	**68.8**	**0.846**	**<0.001**
**3β,5β,20α-PD/3β,5β-THA, conjugates**		**>215**	**93.3**	**68.8**	**0.854**	**<0.001**
^c^***I***_***A/P***_		**<0.128**	**93.3**	**81.3**	**0.904**	**<0.001**

^a^Only those variables having an AUC at significance levels p<0.1 are shown;

^b^the null hypothesis is that AUC = 0.5…no discrimination (AUC = 1…absolute discrimination);

^c^a composite index of ratios of sulfated 3α/β-hydroxy-5α/β-androstane-17-ones to conjugated 5α/β-pregnane-3α/β, 20α-diols (for details see the section Calculations and data analysis)

Of the steroids, 17-hydroxypregnenolone sulfate, conjugated 3α,5α,20α-PD and 3α,5α,17β-AD showed higher levels in the ICP patients, while the 16α-hydroxy-steroids such as 16α-hydroxy-DHEA and 16α-hydroxyandrostenedione were lower in these patients, as were the 17-oxo C19 5α-reduced metabolite 3β,5α-THA and 5β-reduced steroids (3α,5β-THP, 3β,5β-THP, 3α,5β-THA, and 3β,5β-THA).

Table 4 illustrates the associations between ICP as a predicted variable and relevant predictors including steroids measured by RIA and GC-MS as evaluated by OPLS and multiple regression. Although the conjugates of 3β,5α-THA, 3α,5β-THA, and 3α,5α,17β-AD were relevant as predictors, when considering their significance for VIP statistics (p<0.05), the sulfates of 3β,5α-THA and 3α,5β-THA did not reach significance for component loadings of the predictive component (p>0.05). While conjugated pregnanediols (conjugated 3α,5α,20α-PD, conjugated 3α,5β,20α-PD, conjugated 3β,5β,20α-PD, conjugated 3β,5α,20α-PD), and 17-hydroxypregnenolone sulfate were significantly higher in ICP patients, the 20α-dihydropregnenolone, 16α-hydroxy-steroids (16α-hydroxy-DHEA, 16α-hydroxyandrostenedione, and 16α-hydroxyestrone), sulfated 17-oxo-C19-steroids (3α,5α-THA sulfate, 3β,5β-THA sulfate), and 5β-reduced steroids (3α,5β-THP, 3β,5β-THP, 3α,5β-THA) were significantly lower in women with ICP. Table 5 shows the associations between ICP as a predicted variable and relevant steroids measured by GC-MS as predictors using OPLS and multiple regression. Conjugated 3α,5α,17β-AD, 3β,5α-THA sulfate, and 3α,5β-THA sulfate were relevant as predictors when considering their significance in the VIP statistics (p<0.05) but did not reach significance for component loadings of the predictive component (p>0.05).

**Table 4:** Relationships between ICP and circulating steroids measured by RIA (17-hydroxypregnenolone sulfate) and GC-MS (remaining steroids) as evaluated by OPLS model (for details see Statistical analysis).

			OPLS predictive component component loadings (P)				Multiple regression regression coefficients (r)		
		Variable	P	t-statistic	R^a^		r	t-statistic	
Relevant predictors(matrix **X**)		(Gestational age)^1^	0.021	0.14	0.041		-0.018	-0.47	
	↑	(Gestational age)^2^	0.308	2.93	0.608	*	0.135	4.20	**
	↓	20α-Dihydropregnenolone	-0.211	-2.09	-0.416	*	-0.094	-2.44	*
	↑	17-Hydroxypregnenolone sulfate	0.278	3.08	0.548	**	0.123	2.39	*
	↓	16α-Hydroxy-DHEA	-0.265	-6.39	-0.523	**	-0.095	-6.59	**
	↓	16α-Hydroxyandrostenedione	-0.217	-7.73	-0.428	**	-0.086	-3.43	**
	↓	16α-Hydroxyestrone	-0.225	-4.90	-0.444	**	-0.082	-3.71	**
	↓	Pregnanolone (3α,5β-THP)	-0.230	-2.49	-0.453	*	-0.122	-4.26	**
	↓	Epipregnanolone (3β,5β-THP)	-0.291	-2.71	-0.573	*	-0.119	-3.18	**
	↑	Conjugated 5α-pregnane-3α,20α-diol	0.302	4.19	0.601	**	0.146	5.95	**
	↑	Conjugated 5α-pregnane-3β,20α-diol	0.212	2.16	0.422	*	0.075	2.54	*
	↑	Conjugated 5β-pregnane-3α,20α-diol	0.244	2.69	0.485	*	0.082	3.80	**
	↑	Conjugated 5β-pregnane-3β,20α-diol	0.165	1.93	0.328	*	0.054	2.65	*
	↓	Androsterone (3α,5α-THA) sulfate	-0.174	-2.36	-0.345	*	-0.087	-3.10	**
	↓	Epiandrosterone (3β,5α-THA) sulfate	-0.201	-1.74	-0.399		-0.117	-3.67	**
	↓	Etiocholanolone (3α,5β-THA)	-0.265	-5.86	-0.522	**	-0.090	-3.09	**
	↓	Etiocholanolone sulfate	-0.133	-1.40	-0.265		-0.093	-3.05	**
	↓	Epietiocholanolone (3β,5β-THA) sulfate	-0.311	-2.58	-0.617	*	-0.144	-3.17	**
	↑	Conjugated 5α-androstane-3α,17β-diol	0.179	1.67	0.355		0.072	2.07	*
Predicted variable (**Y**)		Intrahepatic cholestasis, LLR^b^	1.000	16.66	0.871	**			
**Explained variability**			87.1% (84.4% after cross-validation)						

^a^R…Component loadings expressed as a correlation coefficients with predictive component,

*p<0.05,

**p<0.01;

^b^LLR…Logarithm of likelihood ratio (logarithm of the ratio of probability that the subject is a patient to probability that the subject is a control); Relationship between LLR of ICP and gestational age is a polynomial of the 2^nd^ order

**Table 5:** Relationships between ICP and circulating steroids measured exclusively by GC-MS, as evaluated by the OPLS model and by multiple regression (for details see Statistical analysis).

			OPLS predictive component component loadings (P)				Multiple regression regression coefficients (r)		
		Variable	P	t-statistic	R^a^		r	t-statistic	
Relevant predictors(matrix **X**)		(Gestational age)^1^	0.013	0.08	0.027		-0.018	-0.47	
	↑	(Gestational age)^2^	0.317	2.82	0.625	*	0.134	3.77	**
	↓	20α-Dihydropregnenolone	-0.204	-2.15	-0.403	*	-0.094	-2.53	*
	↑	20α-Dihydropregnenolone sulfate	0.212	1.74	0.420		0.065	2.10	*
	↓	16α-Hydroxy-DHEA	-0.237	-5.29	-0.467	**	-0.094	-6.32	**
	↓	16α-Hydroxyandrostenedione	-0.206	-7.29	-0.406	**	-0.085	-3.53	**
	↓	16α-Hydroxyestrone	-0.199	-3.69	-0.394	**	-0.082	-3.84	**
	↓	Pregnanolone (3α,5β-THP)	-0.229	-2.27	-0.452	*	-0.122	-4.12	**
	↓	Epipregnanolone (3β,5β-THP)	-0.294	-2.70	-0.581	*	-0.118	-3.41	**
	↑	Conjugated 5α-pregnane-3α,20α-diol	0.319	3.85	0.632	**	0.147	4.37	**
	↑	Conjugated 5α-pregnane-3β,20α-diol	0.227	2.10	0.450	*	0.076	2.61	*
	↑	Conjugated 5β-pregnane-3α,20α-diol	0.261	2.75	0.517	*	0.083	3.54	**
	↑	Conjugated 5β-pregnane-3β,20α-diol	0.180	1.98	0.356	*	0.055	2.46	*
	↓	Androsterone (3α,5α-THA) sulfate	-0.177	-2.12	-0.351	*	-0.088	-3.30	**
	↓	Epiandrosterone (3β,5α-THA) sulfate	-0.194	-1.58	-0.385		-0.117	-3.30	**
	↓	Etiocholanolone (3α,5β-THA)	-0.267	-4.97	-0.528	**	-0.090	-2.86	*
	↓	Etiocholanolone sulfate	-0.132	-1.39	-0.262		-0.093	-2.66	*
	↓	Epietiocholanolone (3β,5β-THA) sulfate	-0.304	-2.44	-0.602	*	-0.144	-3.01	**
	↑	Conjugated 5α-androstane-3α,17β-diol	0.179	1.83	0.355		0.068	2.09	*
	↑	Conjugated 5β-androstane-3α,17β-diol	0.199	2.04	0.394	*	0.073	2.14	*
Predicted variable (**Y**)		Intrahepatic cholestasis, LLR^b^	0.317	2.82	0.625	*			
**Explained variability**		76.9% (70.4% after cross-validation)							

^a^R…Component loadings expressed as a correlation coefficients with predictive component,

*p<0.05,

**p<0.01;

^b^LLR…Logarithm of likelihood ratio (logarithm of the ratio of probability that the subject is a patient to probability that the subject is a control); Relationship between LLR of ICP and gestational age is a polynomial of the 2^nd^ order

While conjugated pregnanediols and conjugated 3α-hydroxy-5α/β-androstane-17β-diols were significantly higher in ICP patients, 20α-dihydropregnenolone, 5β-reduced-20-oxo-pregnanes (3α,5β-THP and 3β,5β-THP), 16α-hydroxy-steroids (16α-hydroxy-DHEA, 16α-hydroxyandrostenedione, and 16α-hydroxyestrone), and three 17-oxo-C19-steroids (3α,5β-THA, 3α,5α-THA sulfate, and 3β,5β-THA sulfate) were significantly lower in women with ICP. In addition, the ratios of conjugated 5α/β-pregnane-3α/β, 20α-diols to the corresponding 3α/β-hydroxy-5α/β-androstane-17-ones were significantly and consistently higher (Table 2), and ROC showed a good discrimination between ICP patients and controls (Table 3). Finally, the values of *I*_*A/P*_ summarizing the lower levels of sulfated 3α/β-hydroxy-5α/β-androstane-17-ones but elevated levels of conjugated 5α/β-pregnane-3α/β, 20α-diols in ICP patients were pronouncedly lower in ICP patients, as demonstrated by the linear model (Table 2) and ROC *I*_*A/P*_ showing high discrimination between these groups (specificity = 93.3%, sensitivity = 81.3%, AUC = 0.904, p<0.001, cut-off value < 0.128 for patients) (see also Table 3).

## Discussion

### The altered steroid metabolome in the circulation of women with ICP

BAs are generally recognized as substances that ultimately threaten the fetus in women with ICP. As documented by our data and by the results of others, there are several common metabolic steps in the biosynthesis of steroids and BAs that are tightly interconnected [43]. It is thus likely that altered steroidogenesis in women with ICP reflects an altered synthesis and metabolism of BAs. Therefore, our results enable us to elucidate the probable associations of individual steroids and specific steps in steroid metabolism with the pathophysiology of ICP.

#### ICP is associated with increased steroid C17-hydroxylation as well as the production of conjugated pregnanediols

A substantial proportion of the precursors of maternal steroids (particularly the female sex hormones and their metabolites) originate in the fetal zone of the fetal adrenal. The activity of this zone is controlled by placental corticoliberin (CRH). In humans and great apes, CRH expression exponentially rises with approaching term [5,44–47]. Interestingly, the manifestation of ICP is most common in the third trimester, and the risk of ICP for the fetus increases with increasing GA. Furthermore, a recent study by Zhou *et al*. [48] reported lower CRH expression in the ICP placenta with an unchanged placental expression of mRNA for both CRH and type 1 CRH receptors (CRH-R1). In addition, they reported that the increase of circulating CRH levels in the ICP group was slower than in controls, particularly in late pregnancy [48].

Therefore, one might expect lower levels of DHEAS and its products testosterone, estradiol and androgen 5α/β–reduced catabolites due to attenuated CYP17A1 activity in the C17,20 lyase step, which is specific for the adrenal *zona reticularis*. However, neither C21 nor C19 Δ^4^ steroids nor estrogens significantly differed between ICP patients and controls, except for lower 16α-hydroxyestrone levels in ICP patients (probably due to reduced 16α-hydroxylase activity as discussed below). This absence of differences in most estrogens is in accordance with the data from Laatikainen *et al*. [49]. Although these authors observed significantly lower levels of DHEA sulfate in ICP patients with delivery at week 38–41 of gestation, they did not find such a difference in ICP patients with delivery at week 35–37 of gestation (with GA most closely resembling our patients).

**Fig 2:**
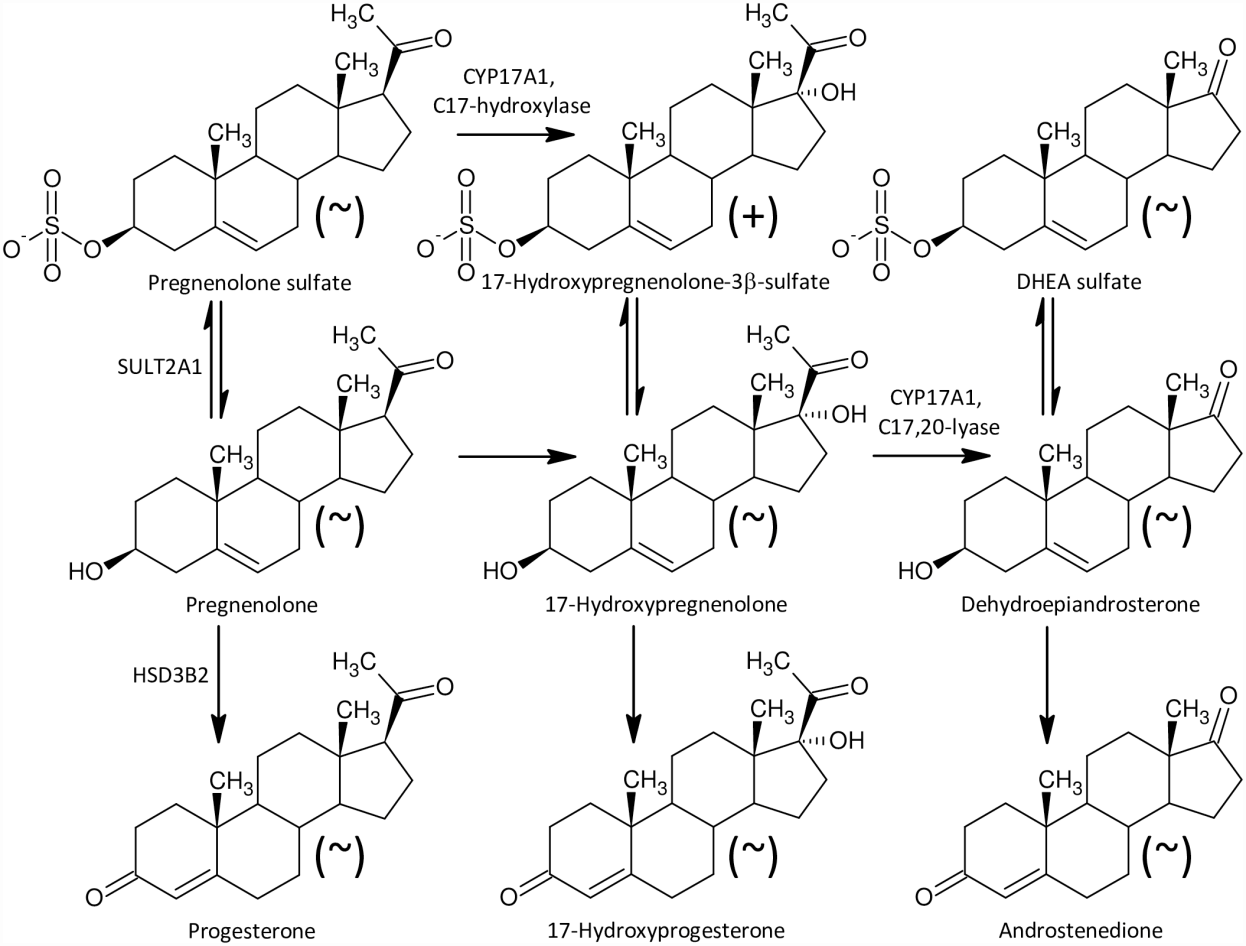
A simplified scheme of steroidogenesis in the Δ^5^ and Δ^4^ metabolic pathways in humans. The symbols in parentheses “+” and “~” represent increased and unchanged steroid levels, respectively, in the ICP group.

In contrast to the lack of differences between our studied group in the C19 Δ^4^ and Δ^5^ steroids (Fig 2), we observed significantly reduced levels of sulfated 17-oxo-C19-5α/β-reduced steroids, which may originate from androstenedione, the levels of which did not differ, however.

**Fig 3:**
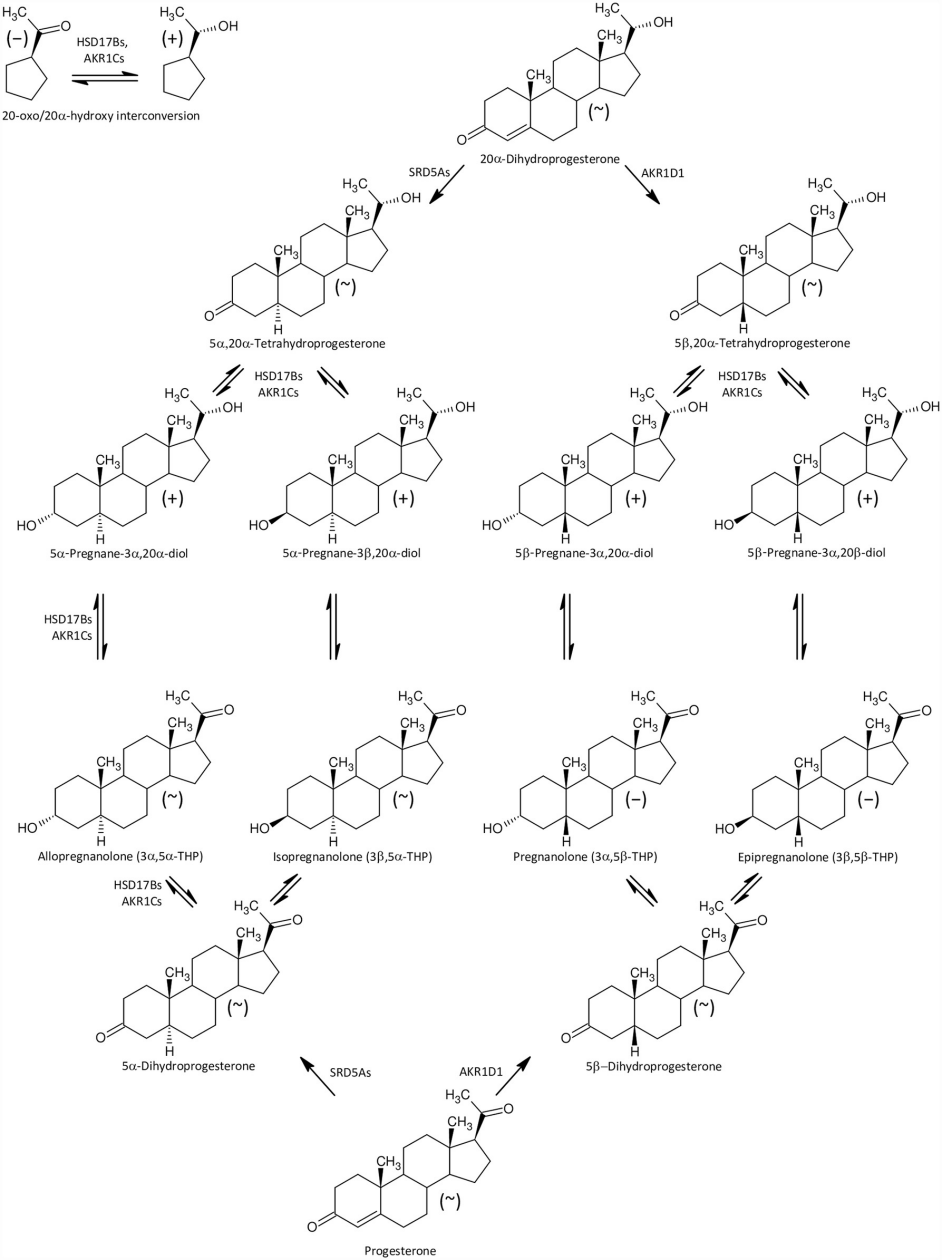
A simplified scheme of 5α/β-reductive progesterone and 20α-dihydroprogesterone catabolism and interconversion between the steroid 20-oxo and 20α-hydroxy-counterparts. The symbols in parentheses “+”, “~”, and ”−” represent increased, unchanged steroid and decreased steroids levels, respectively, in the ICP group.

**Fig 4:**
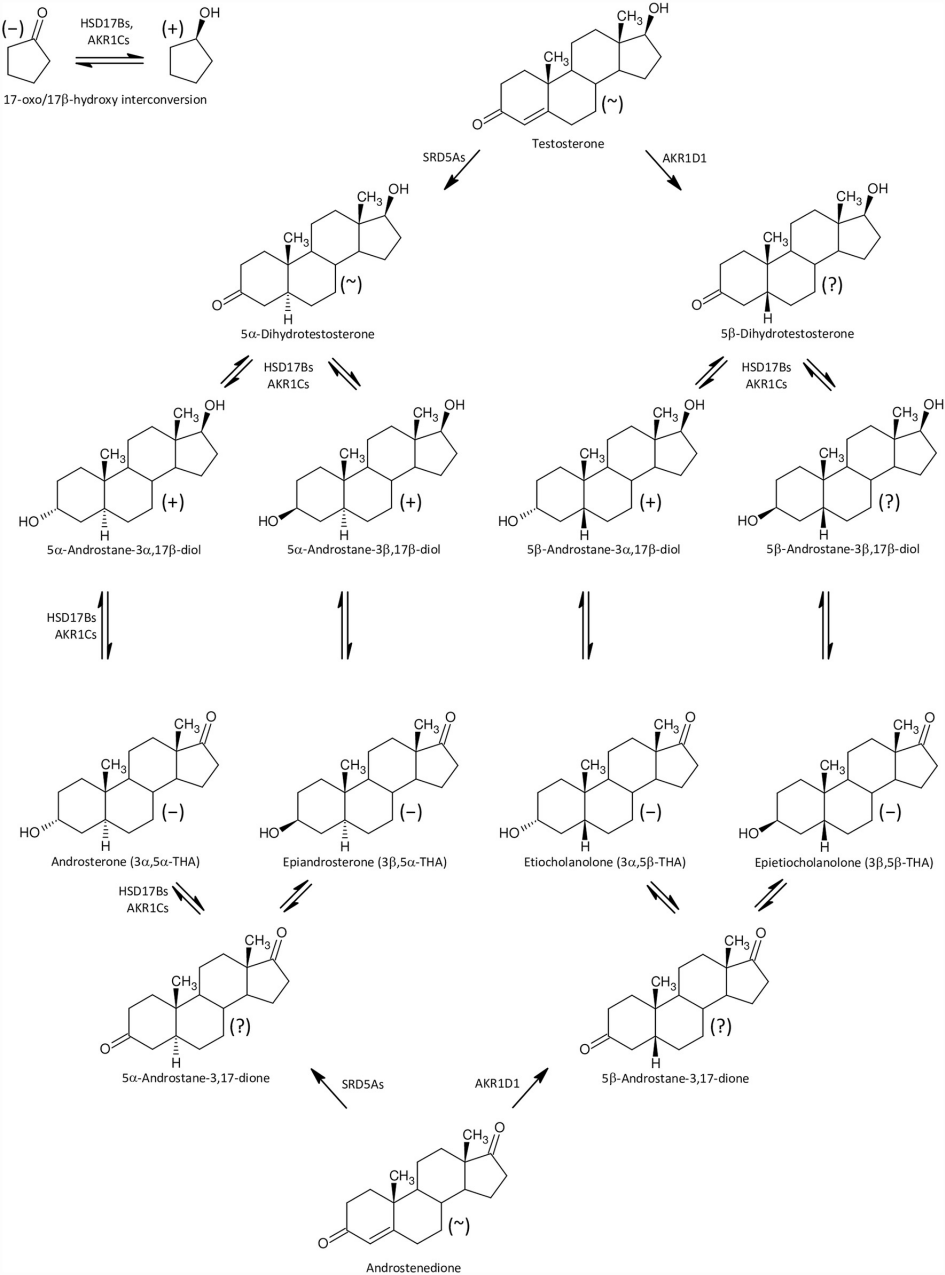
A simplified scheme of 5α/β-reduced C19 steroid biosynthesis in the human “frontdoor pathway”. The symbols in parentheses “+”, “~”, ”−“, and ”?” represent increased, unchanged, decreased and unknown steroids levels, respectively, in the ICP group.

**Fig 5:**
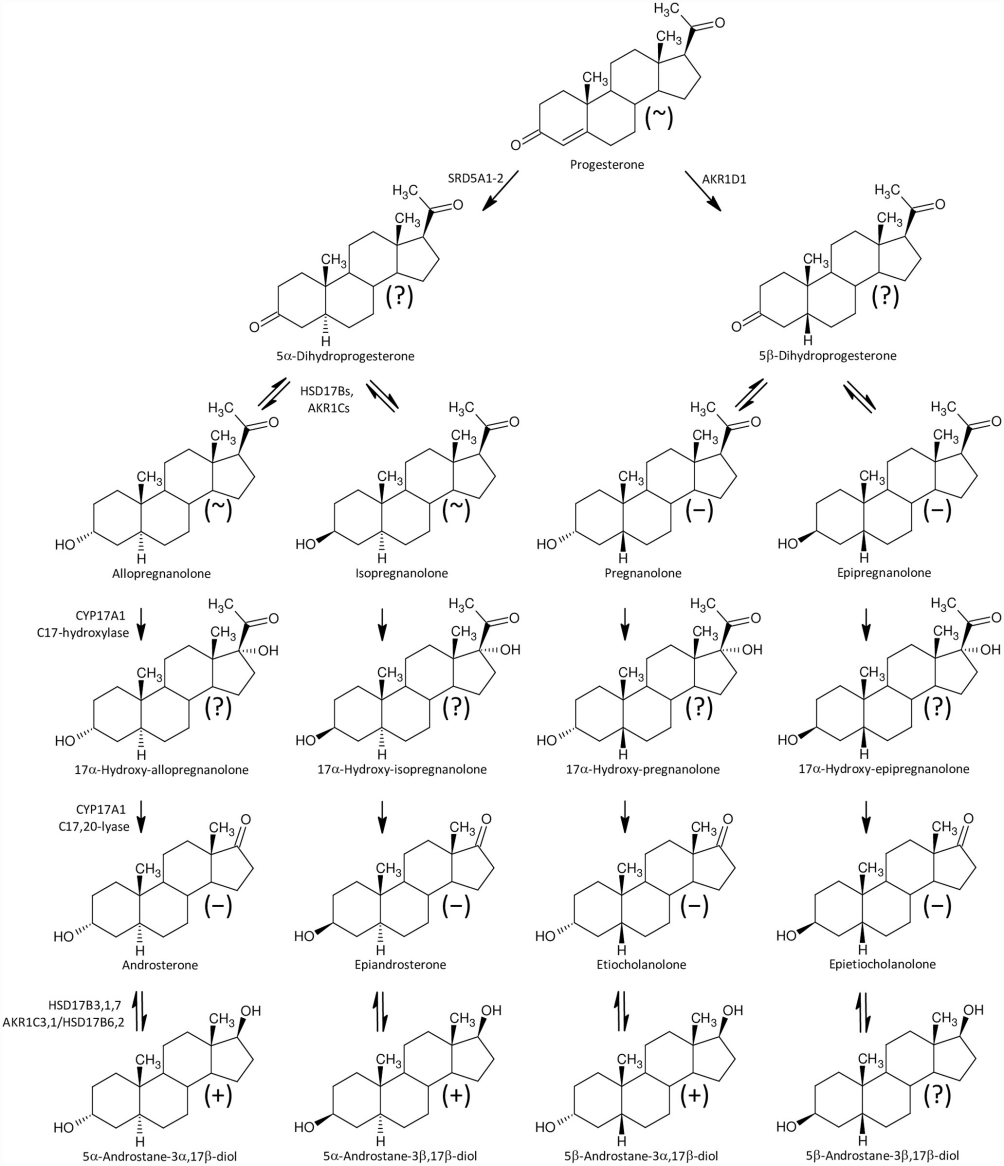
A simplified scheme of 5α/β-reduced C19 steroid biosynthesis in human, “backdoor pathway”. The symbols in parentheses “+”, “~”, ”−“, and ”?” represent increased, unchanged, decreased and unknown steroids levels, respectively, in the ICP group.

Furthermore, besides higher levels of 17-hydroxypregnenolone sulfate (Fig 2) we also found significantly increased concentrations of conjugated pregnanediols (Fig 3), which indicates increased CYP17A1 activity in the C17-hydroxylase step. Perhaps a block at the CYP17A1 lyase step might also contribute to the increased production of 17-hydroxypregnenolone sulfate (Fig 2), as pregnenolone sulfate in the CYP17A1 C17-hydroxylase step transforms directly to 17-hydroxypregnenolone sulfate without previous desulfation. On the other hand, the further (CYB5-induced) scission of the 17,20-carbon-carbon bond only proceeds in unconjugated 17-hydroxypregnenolone, forming unconjugated DHEA, which is then converted to the resulting DHEAS by sulfotransferase SULT2A1 [50,51]. However, in addition to the classical androstenedione and testosterone catabolism to their 5α/β-reduced metabolites (Fig 4) there may be also an alternative “backdoor” steroid metabolism pathway, which was first reported in marsupial gonads but possibly functions in humans as well. CYP17A1 in the major “frontdoor” pathway converts the steroids in the sequence: C21-Δ^5^ 17-deoxy-steroids, C21-Δ^5^ 17-hydroxy-steroids, C19-Δ^5^-steroids; and then C19-Δ^5^-steroids are transformed to their Δ^4^-counterparts by HSD3B2. Alternatively, the so-called “backdoor” pathway might concurrently convert the 17-deoxy-20-oxo-5α-reduced progesterone metabolites in the sequence: C21 17-deoxy-20-oxo-5α-reduced progesterone metabolites, C21 17-hydroxy-20-oxo-5α-reduced-equivalents, and 17-oxo-C19 equivalents [52–54]. There is no information, however, whether this mechanism may also work in 5β-reduced steroids (Fig 5).

Provided that placental CRH production is attenuated in ICP patients, both the classical “frontdoor” as well as the alternative “backdoor” pathways should produce less C19 5α-reduced androstane catabolites in the sulfated form, because the sulfated steroids are relatively stable and may therefore reflect alterations in CYP17A1 C17 hydroxylase and C17,20 lyase activities. In accordance with this, we also found substantially increased levels of the conjugated C21 steroid catabolites pregnanediols (which indicates increased CYP17A1 C17 hydroxylase activity like the elevated 17-hydroxypregnenolone sulfate levels) but significantly reduced levels of sulfated 17-oxo-C19-5α-reduced steroids, signifying attenuated activity of the CYP17A1 C17,20 lyase step. Our data concerning the conjugated 5α-pregnanediols are in accordance with those of others [49,55], who have reported substantially elevated levels of these steroids in the disulfate form in ICP patients. These authors, however, did not find significant differences for the conjugated 5β-pregnanediols, in contrast to what we observed.

Our data does not allow us to fully address the role of the placenta in determining steroids levels, as a corresponding placenta mRNA analysis is not included in this study. Nevertheless, the aforementioned outcomes indicate that ICP is associated with increased steroid C17-hydroxylation as well as the production of conjugated pregnanediols, most likely as a result of attenuated C17,20 lyase activity due to reduced placental CRH.

#### Women with ICP have decreased levels of circulating 5β-steroids

**Fig 6:**
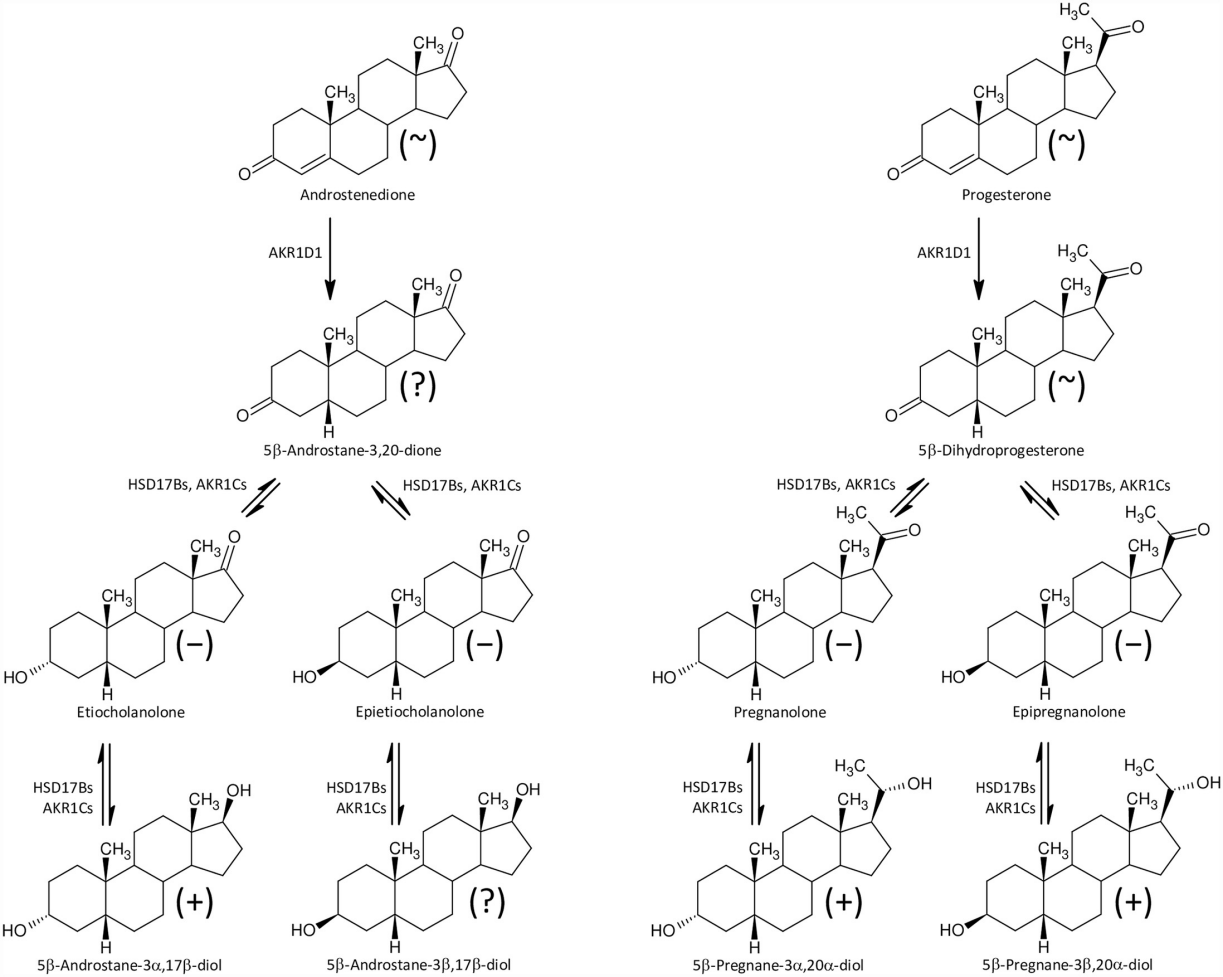
A simplified scheme of the biosynthesis of 5β-reduced C19 and C21 steroids in human. The symbols in parentheses “+”, “~”, ”−“, and ”?” represent increased, unchanged, decreased and unknown steroids levels, respectively, in the ICP group.

The steroid 5β-reductase (AKR1D1) is important in both steroid metabolism and the biosynthesis of BA. AKR1D1 reduces all planar Δ^4^-3-oxo precursors of BA and steroids to their L-shaped 5β-reduced metabolites. An AKR1D1 deficiency results in a BA deficit, the accumulation of hepatotoxic and potentially carcinogenic Δ^4^-3-oxo BA precursors, and an increased production of 5α-BA. Consequently, the aforementioned substances fail to induce the feed-back inhibition of BA biosynthesis via disruption of the farnesoid X-receptor (FXR) [56–58]. Moreover, the Δ^4^-3-oxo BA may attenuate the AKR1D1 activity at relatively low concentrations [59]. In accordance with the data mentioned above, our results indicate reduced AKR1D1 activity in women with ICP, as various unconjugated C21 5β–reduced 20-oxo steroids and both conjugated and unconjugated C19 5β–reduced-17-oxo steroids were lower in these patients (Tables 2–5, Figs 5 and 6). A further adverse consequence of attenuated AKR1D1 activity might be associated with reduced levels of the most abundant fetal GABAergic steroid pregnanolone, which, like the less abundant fetal GABAergic steroid allopregnanolone, may protect the fetal brain from oxidative stress [60,61].

#### ICP is associated with lower serum concentrations of 16α-hydroxy-steroids and impaired xenobiotic removal

Since BAs may be toxic, their concentrations need to be regulated. One of the mechanisms in this regulation proceeds *via* catabolism controlled by CYP3A enzymes including the CYP3A4 and CYP3A7 isoforms [62], which catalyzes the steroid 16α-, 6α/β- and 7β- hydroxylation. These enzymes are primarily expressed in the liver but also in other tissues, and convert of a variety of xenobiotics and endogenous substrates including steroids and BAs to more polar derivatives [62]. As the cytotoxicity of individual BAs is positively associated with their hydrophobicity, their catabolism to more polar catabolites may participate in a feed-forward detoxification mechanism. Toxic compounds including the Δ^4^-3-oxo precursors of BAs stimulate the expression of CYP3A enzymes *via* the pregnane X receptor (PXR), the constitutive androstane receptor (CAR), and the 1,25-dihydroxyvitamin D receptor (VDR), which function as “sensors” [63,64]. Our results support the importance of CYP3A enzymes in the pathophysiology of ICP, as the levels of 16α-hydroxy-steroids tended to have lower values (Tables 2–5), indicating a suppression of an important step of BA detoxification in women suffering from ICP.

### Steroid hormones as factors discriminating ICP patients from controls

A further outcome of this study was the finding of individual predictors and the construction of predictive models for the discrimination of ICP patients from controls. The discrimination for some steroids and particularly for the composite index *I*_*A/P*_ using the ROC was comparable to many of the liver function tests such as liver aminotransferases (see Table 3), total BAs [65,66], and autotaxin [67]. A similar predictivity for pregnanediol disulfates was also demonstrated in the study from Abu Hayyeh et al [55]. Moreover, these authors found a comparable discrimination as in the case of *I*_*A/P*_ (AUC = 0.904, p<0.001, see also Table 3), and even for a combination of pregnanediol disulfates with autotaxin (AUC = 0.910, p<0.001).

**Fig 7:**
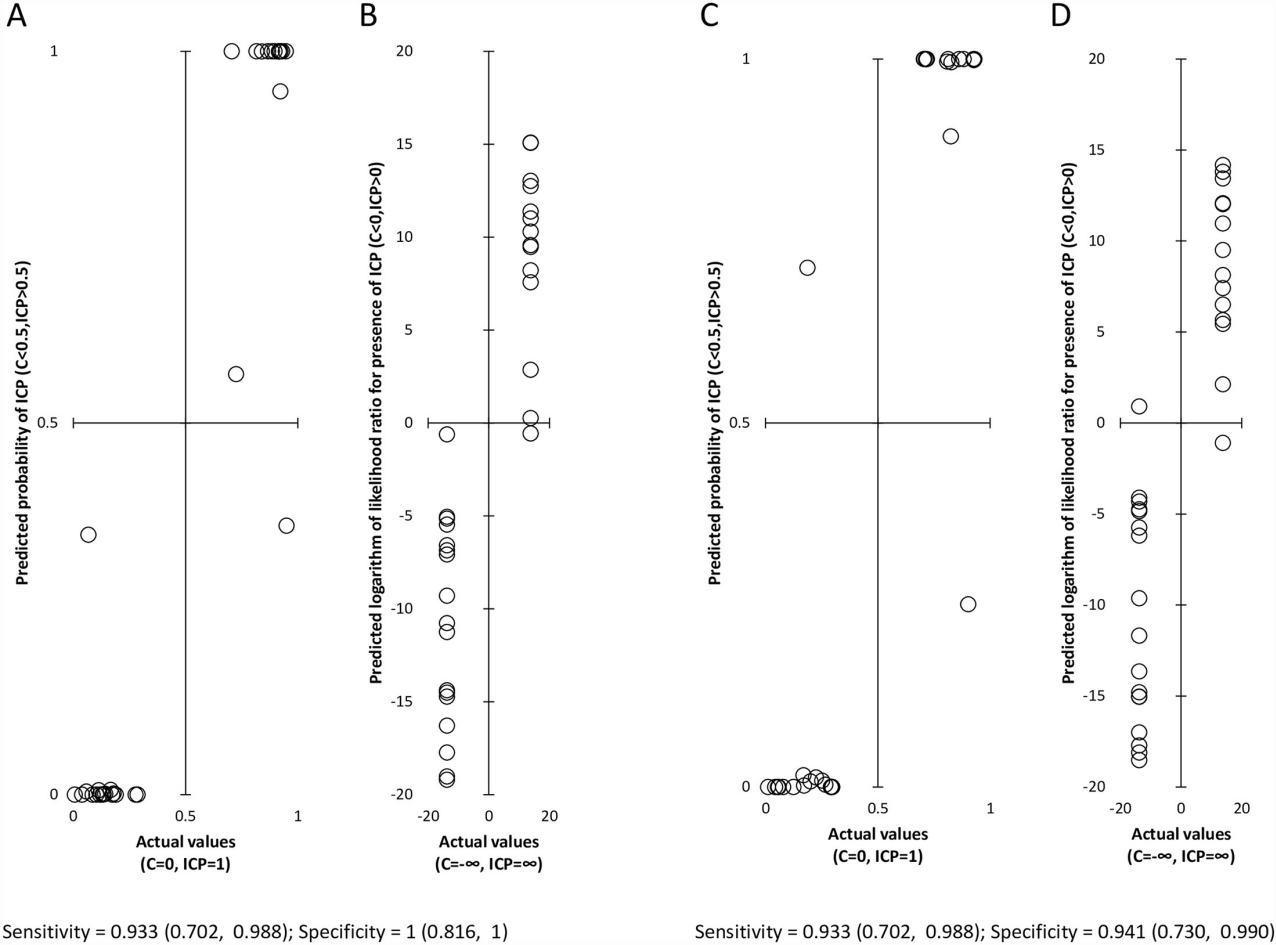
Discrimination of women with intrahepatic cholestasis of pregnancy (ICP) from controls (C) on the basis of maternal circulating steroids measured by radioimmunoassay (17-hydroxypregnenolone sulfate) and GC–MS (remaining steroids) (panels A and B) and on the basis of steroids measured exclusively by GC-MS (panels C and D) using orthogonal projections to latent structures. Panels A and C illustrate the comparison of the probability of pathology with the actual state, while Panels B and D show logarithms of the ratios of the probability that the subject is a patient to the probability that the subject is a control (logarithm of the likelihood ratio, LLR, actual probability of ICP for patients = 1, LLR = ∞, actual probability of ICP for controls = 0, LLR = -∞). A good discrimination using discrimination on the basis of steroids measured by radioimmunoassay and GC–MS (sensitivity of 0.933 (0.702, 0.988) and specificity of 1.000 (0.816, 1.000) (shown as estimates with 95% confidence limits)) as well as for that on the basis of the steroids measured exclusively by GC–MS (sensitivity of 0.933 (0.702, 0.988) and specificity of 0.941 (0.730, 0.990)).

As shown in Fig 7, discriminating between ICP patients and controls using OPLS models was more efficient than discriminating using cut-off values for individual predictors on the basis of ROC analysis. The OPLS model including steroids measured by RIA (17-hydroxypregnenolone sulfate) and GC-MS (remaining steroids) and showed 93.3% (70.2%, 98.8%) sensitivity and 100% (81.6%, 100%) specificity (estimates with 95% confidence limits) (Table 4, Fig 7, panels A and B), and the OPLS model including steroids measured exclusively by GC-MS in a single sample aliquot showed 93.3% (70.2%, 98.8%) sensitivity and 94.1% (73.0%, 99.0%) specificity (Table 5, Fig 7, panels C and D). These data demonstrate that some steroids and particularly their combinations may exhibit a comparable predictivity as the more common ICP markers.

## Conclusions

Although we did not directly evaluate enzyme activities but rather used metabolomic data to qualitatively estimate them, our data indicate physiologically important changes in steroid metabolism associated with ICP, such as increased C17-hydroxylation in the steroid Δ^5^ pathway but an attenuated C17,20 lyase step, which are catalyzed by the same CYP17A1 enzyme. Whereas placental CRH directly stimulates the production of precursors for the synthesis of sex hormones and other bioactive steroids in the placenta, our data are in accordance with the reduced placental CRH levels reported in patients with ICP. Furthermore, we found a lower production of 5β-reduced steroids in the ICP group, presumably because of decreased AKR1D1 activity participating in the synthesis of BAs and lower activities of CYP3A enzymes providing xenobiotic removal.

In addition, we found steroids and particularly steroid combinations that well discriminated between the ICP group and controls. These new data showing the altered steroidogenesis in ICP patients offer more detailed pathophysiological insights into the role of steroids in the development of ICP.

## Supporting Information

S1 Fig Receiver operating characteristic (ROC) for discrimination between ICP patients from controls based on the ratio of conjugated 5α-pregnan-3α,20α-diol (3α,5α,20α-PD) to androsterone (3α,5α-THA) sulfate in the maternal circulation. AUC is the area under the curve and p is the p-value for AUC.
(TIF)

S2 Fig Receiver operating characteristic (ROC) for discrimination between ICP patients from controls based on the ratio of conjugated 5α-pregnan-3β,20α-diol (3β,5α,20α-PD) to epiandrosterone (3β,5α-THA) sulfate in the maternal circulation. AUC is the area under the curve and p is the p-value for AUC.
(TIF)

S3 Fig Receiver operating characteristic (ROC) for discrimination between ICP patients from controls based on the ratio of conjugated 5β-pregnan-3α,20α-diol (3α,5β,20α-PD) to etiocholanolone (3α,5β-THA) sulfate in the maternal circulation. AUC is the area under the curve and p is the p-value for AUC.
(TIF)

S4 Fig Receiver operating characteristic (ROC) for discrimination between ICP patients from controls based on the ratio of conjugated 5β-pregnan-3β,20α-diol (3β,5β,20α-PD) to epietiocholanolone (3β,5β-THA) sulfate in the maternal circulation. AUC is the area under the curve and p is the p-value for AUC.
(TIF)

S5 FigReceiver operating characteristic (ROC) for discrimination between ICP patients from controls based on the composite index *I*_*A/P*_ from the ratios of sulfated 3α/β-hydroxy-5α/β-androstane-17-ones to corresponding conjugated 5α/β-pregnane-3α/β, 20α-diols.$I_{A/P}=\sqrt[{10}]{\frac{\left[3\alpha ,5\alpha -THA,S\right]}{\left[3\alpha ,5\alpha ,20\alpha -PD,C\right]}\cdot \frac{\left[3\beta ,5\alpha -THA,S\right]}{\left[3\beta ,5\alpha ,20\alpha -PD,C\right]}\cdot \frac{\left[3\alpha ,5\beta -THA,S\right]}{\left[3\alpha ,5\beta ,20\alpha -PD,C\right]}\cdot \frac{\left[3\beta ,5\beta -THA,S\right]}{\left[3\beta ,5\beta ,20\alpha -PD,C\right]}}$ [3*α*,5*α*−*THA*,*S*], [3*β*,5*α*−*THA*,*S*], [3*α*,5*β*−*THA*,*S*], and [3*β*,5*β*−*THA*,*S*] are the concentrations of sulfated androsterone, epiandrosterone, etiocholanolone, and epietiocholanolone, respectively and [3*α*,5*α*,20*α*−*PD*,*C*], [3*β*,5*α*,20*α*−*PD*,*C*], [3*α*,5*β*,20*α*−*PD*,*C*], and [3*β*,5*β*,20*α*−*PD*,*C*] are the concentrations of conjugated 5α-pregnan-3α,20α-diol, 5α-pregnan-3β,20α-diol, 5β-pregnan-3α,20α-diol, and 5β-pregnan-3β,20α-diol, respectively (monosulfates + disulfates + glucuronides), respectively, in the maternal circulation. AUC is the area under the curve and p is the p-value for AUC. For details see the sections “Statistical analysis” and “Composite index of ratios of sulfated 3α/β-hydroxy-5α/β-androstane-17-ones to conjugated 5α/β-pregnane-3α/β, 20α-diols for discrimination of ICP patients from controls”.(TIF)

S1 Table Abbreviations for primary data. (XLSX)

S2 Table Primary data. (XLSX)

## References

[1] Pusl T, Beuers U (2007) Intrahepatic cholestasis of pregnancy. Orphanet J Rare Dis 2: 26 17535422 10.1186/1750-1172-2-26 PMC1891276

[2] Perez MJ, Macias RI, Marin JJ (2006) Maternal cholestasis induces placental oxidative stress and apoptosis. Protective effect of ursodeoxycholic acid. Placenta 27: 34–41. 16310035 10.1016/j.placenta.2004.10.020

[3] Simjak P, Parizek A, Vitek L, Cerny A, Adamcova K, Koucky M, et al (2015) Fetal complications due to intrahepatic cholestasis of pregnancy. J Perinat Med 43: 133–139. 10.1515/jpm-2014-0089 25153210

[4] Hill M, Cibula D, Havlikova H, Kancheva L, Fait T, Kancheva R, et al (2007) Circulating levels of pregnanolone isomers during the third trimester of human pregnancy. J Steroid Biochem Mol Biol 105: 166–175. 17583491 10.1016/j.jsbmb.2006.10.010

[5] Hill M, Parizek A, Cibula D, Kancheva R, Jirasek JE, Velikova M, et al (2010) Steroid metabolome in fetal and maternal body fluids in human late pregnancy. J Steroid Biochem Mol Biol 122: 114–132. 10.1016/j.jsbmb.2010.05.007 20580824

[6] Lammert F, Marschall HU, Glantz A, Matern S (2000) Intrahepatic cholestasis of pregnancy: molecular pathogenesis, diagnosis and management. J Hepatol 33: 1012–1021. 11131439 10.1016/s0168-8278(00)80139-7

[7] Reyes H, Sjovall J (2000) Bile acids and progesterone metabolites in intrahepatic cholestasis of pregnancy. Ann Med 32: 94–106. 10766400 10.3109/07853890009011758

[8] Reyes H (2008) Sex hormones and bile acids in intrahepatic cholestasis of pregnancy. Hepatology 47: 376–379. 10.1002/hep.22139 18220280

[9] Glantz A, Reilly SJ, Benthin L, Lammert F, Mattsson LA, Marschall HU, et al (2008) Intrahepatic cholestasis of pregnancy: Amelioration of pruritus by UDCA is associated with decreased progesterone disulphates in urine. Hepatology 47: 544–551. 17968976 10.1002/hep.21987

[10] Meng LJ, Reyes H, Palma J, Hernandez I, Ribalta J, Sjovall J. (1997) Profiles of bile acids and progesterone metabolites in the urine and serum of women with intrahepatic cholestasis of pregnancy. J Hepatol 27: 346–357. 9288610 10.1016/s0168-8278(97)80181-x

[11] Meng LJ, Reyes H, Axelson M, Palma J, Hernandez I, Ribalta J, et al (1997) Progesterone metabolites and bile acids in serum of patients with intrahepatic cholestasis of pregnancy: effect of ursodeoxycholic acid therapy. Hepatology 26: 1573–1579. 9398000 10.1002/hep.510260627

[12] Meng LJ, Reyes H, Palma J, Hernandez I, Ribalta J, Sjovall J (1997) Effects of ursodeoxycholic acid on conjugated bile acids and progesterone metabolites in serum and urine of patients with intrahepatic cholestasis of pregnancy. J Hepatol 27: 1029–1040. 9453429 10.1016/s0168-8278(97)80147-x

[13] Pascual MJ, Serrano MA, El-Mir MY, Macias RI, Jimenez F, Marin JJ (2002) Relationship between asymptomatic hypercholanaemia of pregnancy and progesterone metabolism. Clin Sci (Lond) 102: 587–593. 11980579

[14] Rizzo G, Renga B, Mencarelli A, Pellicciari R, Fiorucci S (2005) Role of FXR in regulating bile acid homeostasis and relevance for human diseases. Curr Drug Targets Immune Endocr Metabol Disord 5: 289–303. 16178789 10.2174/1568008054863781

[15] Abu-Hayyeh S, Papacleovoulou G, Lovgren-Sandblom A, Tahir M, Oduwole O, Jamaludin NA (2013) Intrahepatic cholestasis of pregnancy levels of sulfated progesterone metabolites inhibit farnesoid X receptor resulting in a cholestatic phenotype. Hepatology 57: 716–726. 10.1002/hep.26055 22961653 PMC3592994

[16] Milona A, Owen BM, Cobbold JF, Willemsen EC, Cox IJ, Boudjelal M, et al (2010) Raised hepatic bile acid concentrations during pregnancy in mice are associated with reduced farnesoid X receptor function. Hepatology 52: 1341–1349. 10.1002/hep.23849 20842631

[17] Vallejo M, Briz O, Serrano MA, Monte MJ, Marin JJ (2006) Potential role of trans-inhibition of the bile salt export pump by progesterone metabolites in the etiopathogenesis of intrahepatic cholestasis of pregnancy. J Hepatol 44: 1150–1157. 16458994 10.1016/j.jhep.2005.09.017

[18] Byrne JA, Strautnieks SS, Mieli-Vergani G, Higgins CF, Linton KJ, Thompson RJ (2002) The human bile salt export pump: characterization of substrate specificity and identification of inhibitors. Gastroenterology 123: 1649–1658. 12404239 10.1053/gast.2002.36591

[19] Wang C, Chen X, Zhou SF, Li X (2011) Impaired fetal adrenal function in intrahepatic cholestasis of pregnancy. Med Sci Monit 17: CR265–271. 21525808 10.12659/MSM.881766 PMC3539589

[20] Leslie KK, Reznikov L, Simon FR, Fennessey PV, Reyes H, Ribalta J (2000) Estrogens in intrahepatic cholestasis of pregnancy. Obstet Gynecol 95: 372–376. 10711547 10.1016/s0029-7844(99)00533-5

[21] Imai K, Hayashi Y (1970) Steroid-induced intrahepatic cholestasis in mice. Jpn J Pharmacol 20: 473–481. 5312929 10.1254/jjp.20.473

[22] Martineau M, Papacleovoulou G, Abu-Hayyeh S, Dixon PH, Ji H, Powrie R, et al (2014) Cholestatic pregnancy is associated with reduced placental 11betaHSD2 expression. Placenta 35: 37–43. 10.1016/j.placenta.2013.10.019 24262137

[23] Hill M, Parizek A, Kancheva R, Duskova M, Velikova M, Kriz L, et al (2010) Steroid metabolome in plasma from the umbilical artery, umbilical vein, maternal cubital vein and in amniotic fluid in normal and preterm labor. J Steroid Biochem Mol Biol 121: 594–610. 10.1016/j.jsbmb.2009.10.012 19897033

[24] Hill M, Zarubova J, Marusic P, Vrbikova J, Velikova M, Kancheva R, et al (2010) Effects of valproate and carbamazepine monotherapy on neuroactive steroids, their precursors and metabolites in adult men with epilepsy. J Steroid Biochem Mol Biol 122: 239–252. 10.1016/j.jsbmb.2010.06.003 20541012

[25] Hill M, Vrbikova J, Zarubova J, Kancheva R, Velikova M, Kancheva L, et al (2011) The steroid metabolome in lamotrigine-treated women with epilepsy. Steroids 76: 1351–1357. 10.1016/j.steroids.2011.07.002 21787799

[26] Hill M, Hampl R, Lukac D, Lapcik O, Pouzar V, Sulcova J (1999) Elimination of cross-reactivity by addition of an excess of cross-reactant for radioimmunoassay of 17alpha-hydroxypregnenolone. Steroids 64: 341–355. 10406484 10.1016/s0039-128x(99)00017-3

[27] Vcelakova H, Hill M, Lapcik O, Parizek A (2007) Determination of 17alpha-hydroxypregnenolone sulfate and its application in diagnostics. Steroids 72: 323–327. 17298837 10.1016/j.steroids.2006.11.026

[28] Dehennin L, Peres G (1996) Plasma and urinary markers of oral testosterone misuse by healthy men in presence of masking epitestosterone administration. Int J Sports Med 17: 315–319. 8858400 10.1055/s-2007-972853

[29] Brochu M, Belanger A (1987) Comparative study of plasma steroid and steroid glucuronide levels in normal men and in men with benign prostatic hyperplasia. Prostate 11: 33–40. 2443905 10.1002/pros.2990110105

[30] Sanchez-Guijo A, Oji V, Hartmann MF, Traupe H, Wudy SA (2015) Simultaneous quantification of cholesterol sulfate, androgen sulfates, and progestagen sulfates in human serum by LC-MS/MS. J Lipid Res 56: 1843–1851. 10.1194/jlr.D061499 26239050 PMC4548788

[31] Labrie F, Belanger A, Cusan L, Gomez JL, Candas B (1997) Marked decline in serum concentrations of adrenal C19 sex steroid precursors and conjugated androgen metabolites during aging. J Clin Endocrinol Metab 82: 2396–2402. 9253307 10.1210/jcem.82.8.4160

[32] Brochu M, Belanger A, Dupont A, Cusan L, Labrie F (1987) Effects of flutamide and aminoglutethimide on plasma 5 alpha-reduced steroid glucuronide concentrations in castrated patients with cancer of the prostate. J Steroid Biochem 28: 619–622. 2961945 10.1016/0022-4731(87)90388-8

[33] Tokushige K, Hashimoto E, Kodama K, Tobari M, Matsushita N, Kogiso T, et al (2013) Serum metabolomic profile and potential biomarkers for severity of fibrosis in nonalcoholic fatty liver disease. J Gastroenterol 48: 1392–1400. 10.1007/s00535-013-0766-5 23478936 PMC3889284

[34] Meloun M, Hill M, Militky J, Kupka K (2000) Transformation in the PC-aided biochemical data analysis. Clin Chem Lab Med 38: 553–559. 10987205 10.1515/CCLM.2000.081

[35] Meloun M, Hill M, Militky J, Vrbikova J, Stanicka S, Skrha J (2004) New methodology of influential point detection in regression model building for the prediction of metabolic clearance rate of glucose. Clin Chem Lab Med 42: 311–322. 15080566 10.1515/CCLM.2004.057

[36] Meloun M, Militky J, Hill M, Brereton RG (2002) Crucial problems in regression modelling and their solutions. Analyst 127: 433–450. 12022637 10.1039/b110779h

[37] Escobar-Morreale HF, Asuncion M, Calvo RM, Sancho J, San Millan JL (2001) Receiver operating characteristic analysis of the performance of basal serum hormone profiles for the diagnosis of polycystic ovary syndrome in epidemiological studies. Eur J Endocrinol 145: 619–624. 11720881 10.1530/eje.0.1450619

[38] Czech C, Berndt P, Busch K, Schmitz O, Wiemer J, Most V, et al (2012) Metabolite profiling of Alzheimer's disease cerebrospinal fluid. PLoS One 7: e31501 10.1371/journal.pone.0031501 22359596 PMC3281064

[39] Youden WJ (1950) Index for rating diagnostic tests. Cancer 3: 32–35. 15405679 10.1002/1097-0142(1950)3:1<32::aid-cncr2820030106>3.0.co;2-3

[40] Trygg J, Holmes E, Lundstedt T (2007) Chemometrics in metabonomics. J Proteome Res 6: 469–479. 17269704 10.1021/pr060594q

[41] Trygg J, Wold S (2002) Orthogonal projections to latent structure. J Chemometrics 16: 119–128.

[42] Madsen R, Lundstedt T, Trygg J (2010) Chemometrics in metabolomics—a review in human disease diagnosis. Anal Chim Acta 659: 23–33. 10.1016/j.aca.2009.11.042 20103103

[43] Monte MJ, Marin JJ, Antelo A, Vazquez-Tato J (2009) Bile acids: chemistry, physiology, and pathophysiology. World J Gastroenterol 15: 804–816. 19230041 10.3748/wjg.15.804 PMC2653380

[44] Smith R, Nicholson RC (2007) Corticotrophin releasing hormone and the timing of birth. Front Biosci 12: 912–918. 17127348 10.2741/2113

[45] Smith R, Smith JI, Shen X, Engel PJ, Bowman ME, McGrath SA, et al (2009) Patterns of plasma corticotropin-releasing hormone, progesterone, estradiol, and estriol change and the onset of human labor. J Clin Endocrinol Metab 94: 2066–2074. 10.1210/jc.2008-2257 19258402

[46] Sirianni R, Mayhew BA, Carr BR, Parker CR Jr., Rainey WE (2005) Corticotropin-releasing hormone (CRH) and urocortin act through type 1 CRH receptors to stimulate dehydroepiandrosterone sulfate production in human fetal adrenal cells. J Clin Endocrinol Metab 90: 5393–5400. 16014403 10.1210/jc.2005-0680

[47] Sirianni R, Rehman KS, Carr BR, Parker CR Jr., Rainey WE (2005) Corticotropin-releasing hormone directly stimulates cortisol and the cortisol biosynthetic pathway in human fetal adrenal cells. J Clin Endocrinol Metab 90: 279–285. 15494460 10.1210/jc.2004-0865

[48] Zhou F, He MM, Liu ZF, Zhang L, Gao BX, Wang XD (2013) Expression of corticotrophin-releasing hormone and its receptor in patients with intrahepatic cholestasis of pregnancy. Placenta 34: 401–406. 10.1016/j.placenta.2013.02.006 23478074

[49] Laatikainen TJ, Peltonen JI, Nylander PL (1974) Effect of maternal intrahepatic cholestasis on fetal steroid metabolism. J Clin Invest 53: 1709–1715. 4275337 10.1172/JCI107722 PMC302667

[50] Storbeck KH, Swart AC, Goosen P, Swart P (2013) Cytochrome b5: novel roles in steroidogenesis. Mol Cell Endocrinol 371: 87–99. 10.1016/j.mce.2012.11.020 23228600

[51] Neunzig J, Sanchez-Guijo A, Mosa A, Hartmann MF, Geyer J, Wudy SA, et al (2014) A steroidogenic pathway for sulfonated steroids: the metabolism of pregnenolone sulfate. J Steroid Biochem Mol Biol 144 Pt B: 324–333. 10.1016/j.jsbmb.2014.07.005 25038322

[52] Homma K, Hasegawa T, Nagai T, Adachi M, Horikawa R, Fujiwara I, et al (2006) Urine steroid hormone profile analysis in cytochrome P450 oxidoreductase deficiency: implication for the backdoor pathway to dihydrotestosterone. J Clin Endocrinol Metab 91: 2643–2649. 16608896 10.1210/jc.2005-2460

[53] Kamrath C, Hartmann MF, Wudy SA (2013) Androgen synthesis in patients with congenital adrenal hyperplasia due to 21-hydroxylase deficiency. Horm Metab Res 45: 86–91. 10.1055/s-0032-1331751 23345132

[54] Fukami M, Homma K, Hasegawa T, Ogata T (2013) Backdoor pathway for dihydrotestosterone biosynthesis: implications for normal and abnormal human sex development. Dev Dyn 242: 320–329. 10.1002/dvdy.23892 23073980

[55] Abu-Hayyeh S, Ovadia C, Lieu T, Jensen DD, Chambers J, Dixon PH, et al (2015) Prognostic and mechanistic potential of progesterone sulfates in intrahepatic cholestasis of pregnancy and pruritus gravidarum. Hepatology. 10.1002/hep.28265 PMC4869673 26426865

[56] Gonzales E, Cresteil D, Baussan C, Dabadie A, Gerhardt MF, Jacquemin E (2004) SRD5B1 (AKR1D1) gene analysis in delta(4)-3-oxosteroid 5beta-reductase deficiency: evidence for primary genetic defect. J Hepatol 40: 716–718. 10.1016/j.jhep.2003.12.024 15030995

[57] Chen M, Jin Y, Penning TM (2015) In-Depth Dissection of the P133R Mutation in Steroid 5beta-Reductase (AKR1D1): A Molecular Basis of Bile Acid Deficiency. Biochemistry 54: 6343–6351. 10.1021/acs.biochem.5b00816 26418565 PMC4696769

[58] Lemonde HA, Custard EJ, Bouquet J, Duran M, Overmars H, Scambler PJ, et al (2003) Mutations in SRD5B1 (AKR1D1), the gene encoding delta(4)-3-oxosteroid 5beta-reductase, in hepatitis and liver failure in infancy. Gut 52: 1494–1499. 12970144 10.1136/gut.52.10.1494 PMC1773813

[59] Chen M, Drury JE, Penning TM (2011) Substrate specificity and inhibitor analyses of human steroid 5beta-reductase (AKR1D1). Steroids 76: 484–490. 10.1016/j.steroids.2011.01.003 21255593 PMC3056882

[60] Hirst JJ, Kelleher MA, Walker DW, Palliser HK (2014) Neuroactive steroids in pregnancy: key regulatory and protective roles in the foetal brain. J Steroid Biochem Mol Biol 139: 144–153. 10.1016/j.jsbmb.2013.04.002 23669456

[61] Hill M, Paskova A, Kanceva R, Velikova M, Kubatova J, Kancheva L, et al (2014) Steroid profiling in pregnancy: a focus on the human fetus. J Steroid Biochem Mol Biol 139: 201–222. 10.1016/j.jsbmb.2013.03.008 23583279

[62] Cheng J, Ma X, Gonzalez FJ (2011) Pregnane X receptor- and CYP3A4-humanized mouse models and their applications. Br J Pharmacol 163: 461–468. 10.1111/j.1476-5381.2010.01129.x 21091656 PMC3101609

[63] Li T, Chiang JY (2013) Nuclear receptors in bile acid metabolism. Drug Metab Rev 45: 145–155. 10.3109/03602532.2012.740048 23330546 PMC3676171

[64] Chen J, Zhao KN, Chen C (2014) The role of CYP3A4 in the biotransformation of bile acids and therapeutic implication for cholestasis. Ann Transl Med 2: 7 10.3978/j.issn.2305-5839.2013.03.02 25332983 PMC4200650

[65] Huang WM, Gowda M, Donnelly JG (2009) Bile acid ratio in diagnosis of intrahepatic cholestasis of pregnancy. Am J Perinatol 26: 291–294. 10.1055/s-0028-1103158 19021098

[66] Chen J, Deng W, Wang J, Shao Y, Ou M, Ding M (2013) Primary bile acids as potential biomarkers for the clinical grading of intrahepatic cholestasis of pregnancy. Int J Gynaecol Obstet 122: 5–8. 10.1016/j.ijgo.2013.02.015 23562588

[67] Kremer AE, Bolier R, Dixon PH, Geenes V, Chambers J, Tolenaars D, et al (2015) Autotaxin activity has a high accuracy to diagnose intrahepatic cholestasis of pregnancy. J Hepatol 62: 897–904. 10.1016/j.jhep.2014.10.041 25450205

