# Supplementary figures and images for: A Comprehensive Evaluation of Steroid Metabolism in Women with Intrahepatic Cholestasis of Pregnancy
Source: PLoS One. 2016 Aug 5;11(8):e0159203. doi: 10.1371/journal.pone.0159203 (PMC4975406; doi:10.1371/journal.pone.0159203)

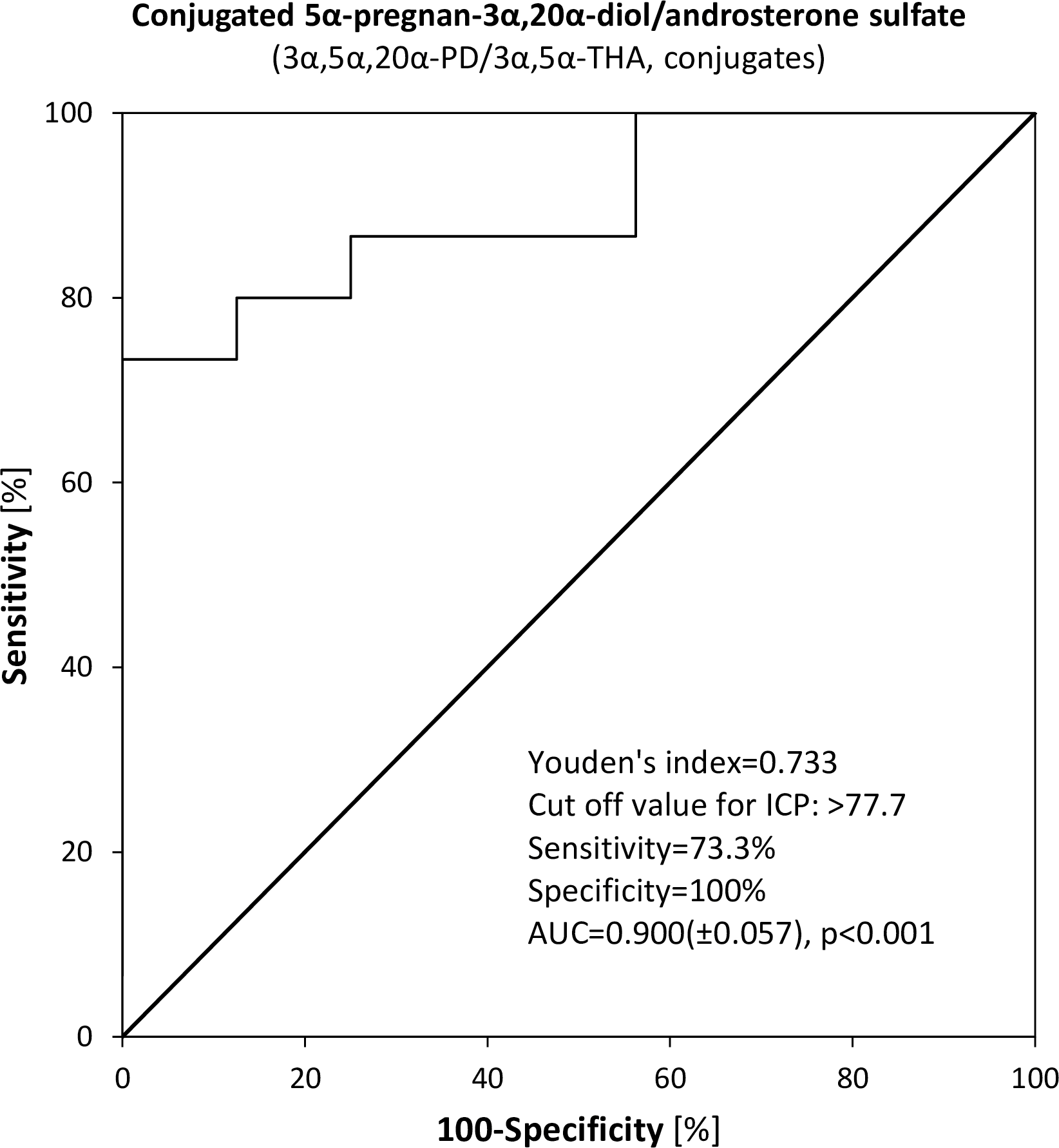

Supplement: S1 Fig — AUC is the area under the curve and p is the p-value for AUC. (TIF) [file pone.0159203.s001.tif]

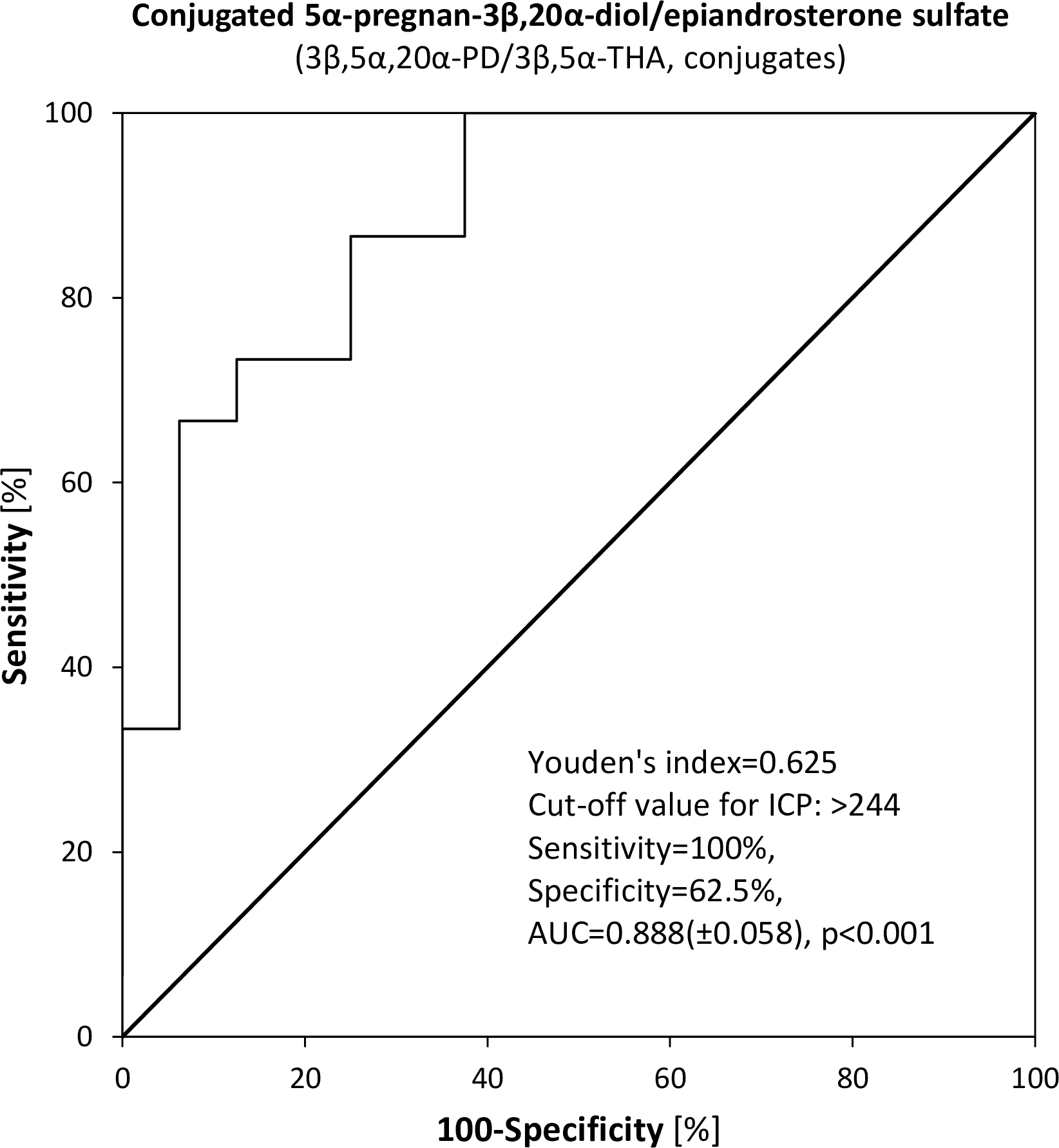

Supplement: S2 Fig — AUC is the area under the curve and p is the p-value for AUC. (TIF) [file pone.0159203.s002.tif]

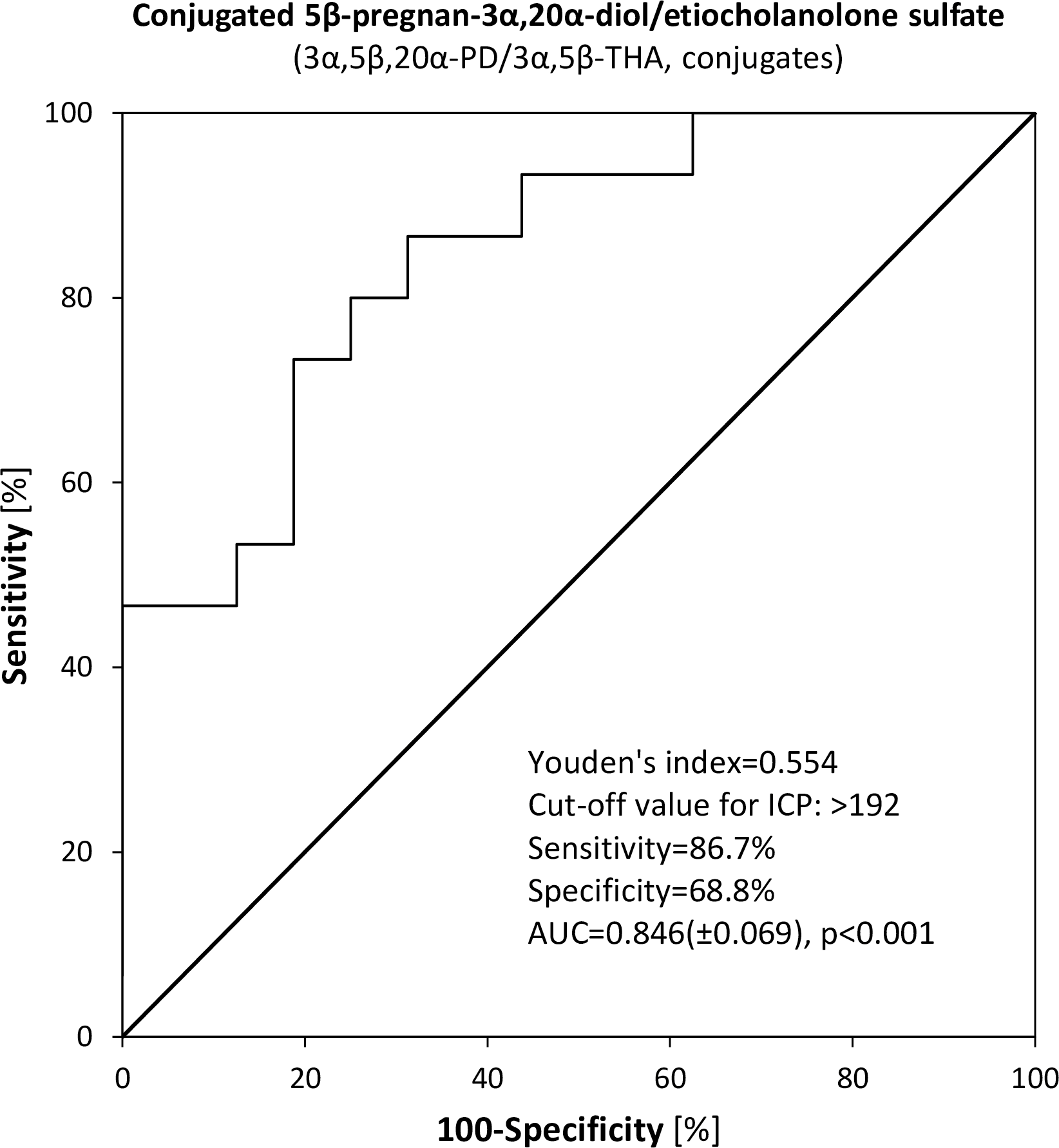

Supplement: S3 Fig — AUC is the area under the curve and p is the p-value for AUC. (TIF) [file pone.0159203.s003.tif]

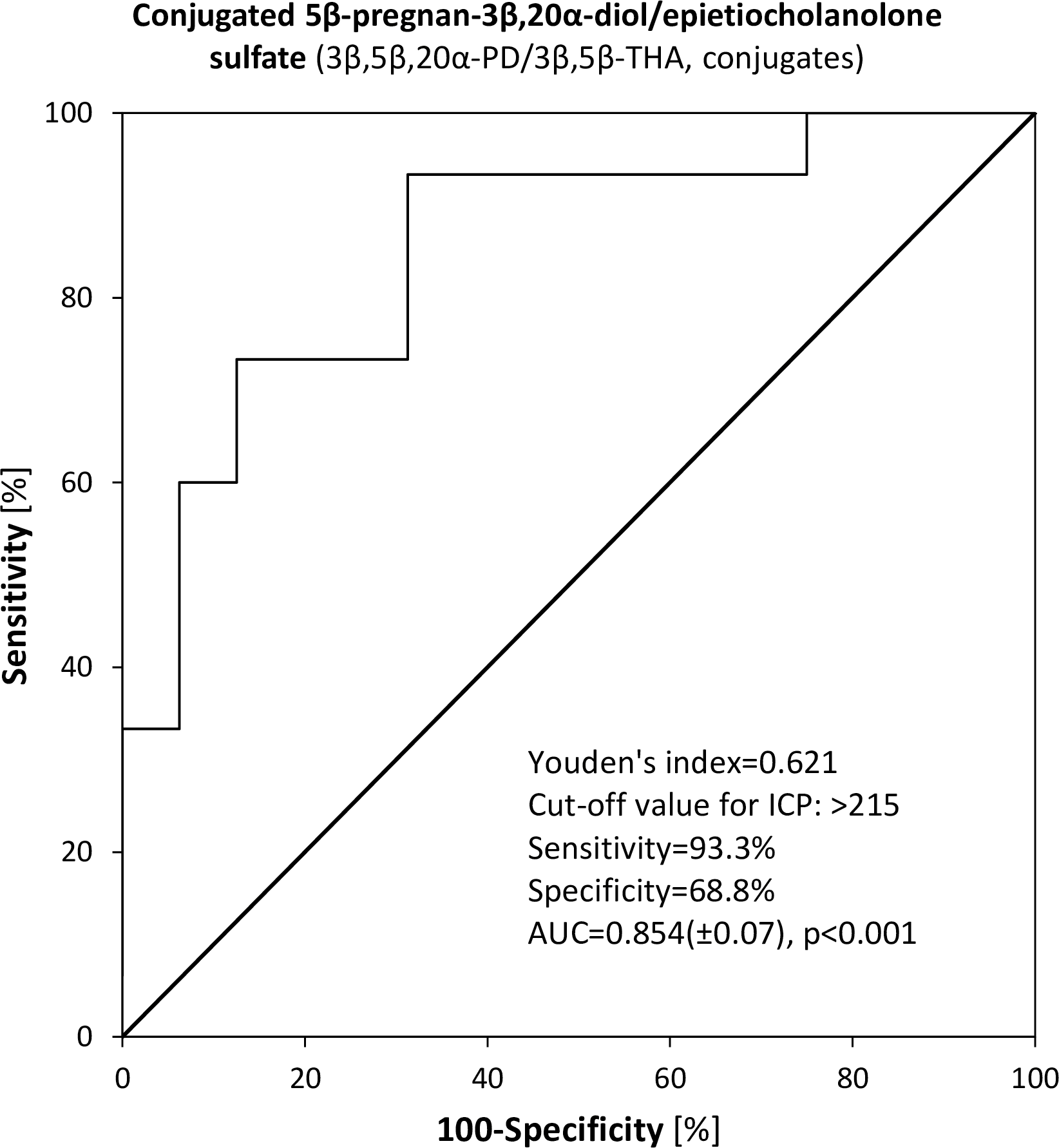

Supplement: S4 Fig — AUC is the area under the curve and p is the p-value for AUC. (TIF) [file pone.0159203.s004.tif]

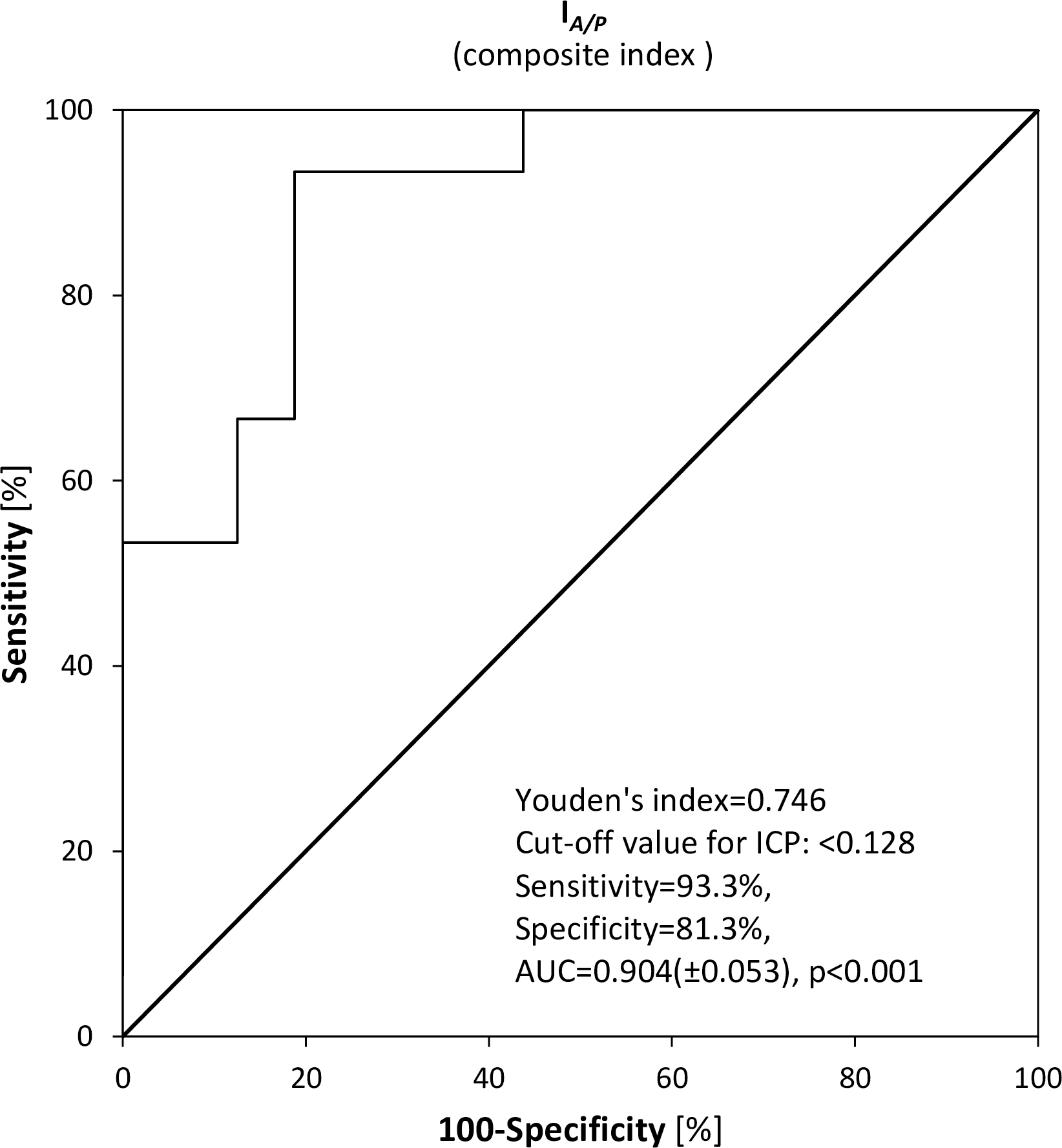

Supplement: S5 Fig — IA/P=[3α,5α−THA,S][3α,5α,20α−PD,C]⋅[3β,5α−THA,S][3β,5α,20α−PD,C]⋅[3α,5β−THA,S][3α,5β,20α−PD,C]⋅[3β,5β−THA,S][3β,5β,20α−PD,C]10 [3α,5α−THA,S], [3β,5α−THA,S], [3α,5β−THA,S], and [3β,5β−THA,S] are the concentrations of sulfated androsterone, epiandrosterone, etiocholanolone, and epietiocholanolone, respectively and [3α,5α,20α−PD,C], [3β,5α,20α−PD,C], [3α,5β,20α−PD,C], and [3β,5β,20α−PD,C] are the concentrations of conjugated 5α-pregnan-3α,20α-diol, 5α-pregnan-3β,20α-diol, 5β-pregnan-3α,20α-diol, and 5β-pregnan-3β,20α-diol, respectively (monosulfates + disulfates + glucuronides), respectively, in the maternal circulation. AUC is the area under the curve and p is the p-value for AUC. For details see the sections “Statistical analysis” and “Composite index of ratios of sulfated 3α/β-hydroxy-5α/β-androstane-17-ones to conjugated 5α/β-pregnane-3α/β, 20α-diols for discrimination of ICP patients from controls”. (TIF) [file pone.0159203.s005.tif]
